# Women are credited less in science than men

**DOI:** 10.1038/s41586-022-04966-w

**Published:** 2022-06-22

**Authors:** Matthew B. Ross, Britta M. Glennon, Raviv Murciano-Goroff, Enrico G. Berkes, Bruce A. Weinberg, Julia I. Lane

**Affiliations:** 1 Department of Economics and School of Public Policy and Urban Affairs, Northeastern University, Boston, MA USA; 2 Wharton School, University of Pennsylvania, Philadelphia, PA USA; 3 National Bureau of Economic Research, Cambridge, MA USA; 4 Questrom School of Business, Boston University, Boston, MA USA; 5 Department of Economics, Ohio State University, Columbus, OH USA; 6 Wagner School of Public Policy, New York University, New York, NY USA

**Keywords:** Economics, Sociology

## Abstract

There is a well-documented gap between the observed number of works produced by women and by men in science, with clear consequences for the retention and promotion of women^1^. The gap might be a result of productivity differences^2–5^, or it might be owing to women’s contributions not being acknowledged^6,7^. Here we find that at least part of this gap is the result of unacknowledged contributions: women in research teams are significantly less likely than men to be credited with authorship. The findings are consistent across three very different sources of data. Analysis of the first source—large-scale administrative data on research teams, team scientific output and attribution of credit—show that women are significantly less likely to be named on a given article or patent produced by their team relative to their male peers. The gender gap in attribution is present across most scientific fields and almost all career stages. The second source—an extensive survey of authors—similarly shows that women’s scientific contributions are systematically less likely to be recognized. The third source—qualitative responses—suggests that the reason that women are less likely to be credited is because their work is often not known, is not appreciated or is ignored. At least some of the observed gender gap in scientific output may be owing not to differences in scientific contribution, but rather to differences in attribution.

## Abstract (web summary)

The difference between the number of men and women listed as authors on scientific papers and inventors on patents is at least partly attributable to unacknowledged contributions by women scientists.

## Main

Gender differences in observed scientific output are well-documented: women both publish and patent less than men^1^. The causes of these differences are not well understood. Analysis using individual data has suggested that women are less productive because they work in less welcoming work environments^2^, have greater family responsibilities^3^, have different positions in the laboratory^4^ or differ in the type of supervision they are provided^5^. Recent work has suggested that women are not less productive, but rather that their work is undervalued^8^. The analysis in this Article uses new data on research teams to suggest that women are accorded less credit than men: they are systematically less likely to be named as authors on articles and patents.

The possibility that women receive less recognition for their scientific contributions is not hypothetical: the canonical example is that of Rosalind Franklin. Franklin’s pivotal contribution to the discovery of the structure of DNA initially went unrecognized^6^, and it was not until long after she died that the scientific community became aware that she was wrongfully denied authorship on the original Crick and Watson paper. Indeed, her contribution was apparently only recognized because Watson’s account of the discovery was so incorrect^9^ and stimulated a reconstruction of events by Franklin’s friends^10^. More recently, Walter Isaacson recounts Jennifer Doudna’s concern that she and Emmanuelle Charpentier were being relegated to “minor players” in the history and commercial use of CRISPR-Cas9^7^. The open questions, of course, are how many women’s contributions have been missed in similar but less high-profile circumstances, and how many women have been discouraged from pursuing careers in science as a result^11^.

Finding ‘what isn’t there’ from ‘what is there’ is a fundamental problem in statistics, and has been used to address such vastly different questions as calculating the return on investment of mutual funds (after accounting for funds that no longer exist) or the optimal placement of armour on aeroplanes in the Second World War^12^ (after accounting for those that did not return). The problem of selecting on the dependent variable is also prevalent in the social sciences; for example, in only observing the labour supply of people who participate in the labour market^13^ or studying the drivers of economic development by selecting a few successful industrializing countries^14^.

The first steps in identifying the missing data in these two examples are to describe the population from which the sample of observations is drawn and then to document the degree of missingness. Subsequent steps then characterize the sources of the missingness. The large-scale bibliometrics databases used to study scientific output consist only of named authors or inventors (not unnamed contributors), and cannot be used to find who is not named; carefully curated case studies are too small to generalize^15^. The unique data on research teams used in this paper are, by contrast, fit for the purpose: they consist of information on 9,778 teams over a four-year period: the 128,859 individuals working in those teams, matched to 39,426 journal articles and 7,675 patents produced by those teams (Methods, ‘Construction of administrative data’). Because the data include information about the positions held by each individual on each team as well as their gender, it is possible to calculate for each individual whether they did or did not receive credit on a given article and to calculate differences by gender.

The evidence generated from the analysis described in this paper suggests that Rosalind Franklin is far from unique in not receiving credit for her work. If credit is defined simply as ever being named an author, women account for only 34.85% of the authors on a team, even though they make up just under half of the workforce (48.25%; Extended Data Table 2). When credit is defined as the likelihood of being listed as an author on a given document (relative to the mean) produced by a research team, there is a 13.24% gap for articles and a 58.40% gap for patents in the likelihood that women are named on any given article or patent produced by their team (Extended Data Table 4, column 5). The chances of women receiving credit on an article decrease by 4.78% relative to the baseline rate of 3.18% (*P* < 0.0001; two-sided *t*-test; test value = −3.8, effect size = −0.0015 percentage points (pp)) for each 1 log point increase in citations (Extended Data Table 7).

The results are confirmed by appealing to a completely different source of quantitative data—a survey of 2,660 scientists regarding the allocation of credit (Methods, ‘Survey design and collection’ and Supplementary Information, part 3). Exclusion from authorship is common and differs significantly by gender: 42.95% of women and 37.81% of men reported that they had been excluded from authorship (*P* = 0.0151; two-sided *t*-test; test value = −2.4327, effect size = −0.0514), and significantly more women (48.97%) than men (39.13%) report that others underestimated their contribution (*P* = 0.0036; two-sided *t*-test; test value = −2.9218, effect size = −0.0984).

Qualitative analysis—open-ended narrative statements by survey respondents as well as personal interviews with consenting authors (approach detailed in Methods, ‘Survey design and collection’ and ‘Qualitative evidence’ and Supplementary Information, part 3)—was also consistent. Authors noted that the rules of credit allocation were frequently unclear and often determined by senior investigators. A complex mix of factors, particularly field, rank, culture and gender, was identified. However, an overarching theme was that the rules governing scientific contributions were often not codified, not understood by all members of the research team, or simply ignored. The necessary level of work required for authorship is often not clear to everyone participating on research teams, and the level of work deemed necessary to receive attribution can vary on the basis of the idiosyncratic personal preferences and a team member’s relationship with the principal investigator (PI). Thus, women and other historically marginalized groups must often put in significantly more effort in order for their scientific contributions to be recognized.

Our analyses on administrative, survey, and qualitative data suggest that even 70 years later, the same factors that led to the denial of Rosalind Franklin’s authorship of the pivotal work on the structure of DNA are still at work. At least some of the observed gender gap in scientific output may not be owing to differences in scientific contribution, but to differences in attribution within research teams.

## Attribution and administrative data

Unpacking the structure of research teams to understand whose work is not recognized requires identifying each individual on each research team, characterizing their position by their job title, and then determining whether or not they are named on the articles and patents produced by the research team. Administrative data can be used to provide highly granular information about who works on which research project because records in human resources both document every payment that is made during each pay period from each grant and provide information on each employee’s job title. Currently, 118 campuses from 36 participating universities provide their deidentified data to the Institute for Research on Innovation and Science at the University of Michigan, which processes and standardizes the information as analytical files^16^. The earliest year for which data were provided by a participating institution was 2000 and the latest was 2019, and the data include information on payments of wages from individual grants to all people employed by each grant, including information on the job title for which a person is paid on a particular grant (Methods, ‘Construction of administrative data’).

Teams were constructed around a central PI, their associated grants, and individuals employed on those grants from 2013–2016. The scientific field of each team is identified by using the title of all associated grants and comparing the grants with a pool of text that describes each scientific field using a ‘wiki-labelling’ approach^17–19^. Scientific documents were linked to a team if the article or patent acknowledged one of the team’s grants and/or any member of the team was listed as an author on that article or patent (further details in Methods, ‘Construction of administrative data’).

Attribution can be measured in many ways using these data. Three measures are constructed for the purposes of this paper: (1) the rate at which individuals are ever named as an author on any scientific document: the ‘ever-author’ rate, (2) the rate at which individuals are named as an author on a given scientific document produced by their team—the ‘attribution’ rate, and (3) the rate at which individuals are named to any given high-impact document—the ‘high-impact attribution’ rate (Methods, ‘Analytical sample’).

The first and simplest measure is the ever-author rate, which characterizes an individual as an author if he or she was ever named as an author or an inventor during the analysis period. As shown in Table 1, 16.97% of individuals are classified as authors using this measure, but the probability that men are ever named is 21.17% whereas the probability for women is 12.15%. Table 1 also shows that there are two reasons for this gap: the junior positions of women in research teams, and under-representation in attribution given their position. First, women are less likely to be in the senior positions that are associated with ever being named an author, 'ever authorship'. The highest ever authorship rate (45.70%) is for faculty members, yet only 11.30% of women (versus 19.72% of men) in the sample are faculty members. Conversely, the ‘ever authorship’ rate for research staff is 8.63%, yet 47.81% of women are research staff, compared with 28.73% of men. Second, holding the distribution of positions constant (at the grand means), women are 4.82% less likely to ever be named as authors. In the case of graduate students, for example, 14.97% of women are ever named as an author on a document compared with 21.37% of men. The consequences of such disparities on the retention of senior women in and the attraction of young women to scientific careers are unlikely to be positive.

**Table 1:** Gender differences in position and ‘ever authorship’

Job title	Frequency of job title in the full sample			Likelihood of ever receiving attribution		
	Total	Women	Men	Total	Women	Men
Faculty	14.85%	11.30%	19.72%	45.70%	41.25%	48.86%
Postdoc	8.63%	6.00%	9.08%	25.17%	22.35%	27.31%
Graduate student	24.15%	17.42%	25.06%	18.69%	14.97%	21.37%
Research staff	35.41%	47.81%	28.73%	8.63%	6.59%	11.01%
Undergraduate	16.96%	17.48%	17.42%	2.61%	2.22%	3.10%
Total/average	100%	100%	100%	16.97%	12.15%	21.17%

This table provides descriptive statistics that show the percentage of employees who worked in university research teams between 2013 and 2016 (left three columns), as well as those who appeared on at least one scientific document published from 2014 to 2016 as an author or inventor (right three columns). The percentages are computed over the 128,859 unique employees in the dataset. The totals include men, women and those whose gender was not imputed. Further details are provided in Extended Data Table 1 and Methods, ‘Construction of administrative data’.

Although illustrative, the ever-author rate does not fully capture differential attribution. In our motivating example, Franklin could have been named as an author on some articles or patents emanating from the research team other than the DNA paper with Crick and Watson. The second authorship measure is the attribution rate, which represents the likelihood that a woman receives credit on a given scientific document produced by her research team.

The empirical implementation of what is a relatively straightforward conceptual framework is more difficult, but the data are rich enough to allow such calculations (see Methods, ‘Analytical sample’ for details). The denominator—the set of ‘potential authorships’—was created by associating all members of each team who were employed one year before the publication or application date to all associated articles or patents emanating from that team during the analysis period. Since some individuals, such as research staff, are on multiple teams, they are proportionately allocated across teams using a set of analytical weights (Methods, ‘Analytical sample’). The numerator—attribution—was defined as ‘actual authorships’ on those articles and patents. Thus, the attribution rate is the ratio of actual authorships to potential authorships. The overall attribution rate for any team member on either a patent or article is 3.2%. On average across all job titles and fields, women have a 2.12% probability of being named on any scientific document, whereas men are twice as likely to be named (4.23%) (*P* = 0.0000; two-sided *t*-test; test value = 19.5823, effect size = 2.11%; Extended Data Tables 2 and 3).

The data are rich enough to examine whether the observed gender gap simply reflects gender differences in organizational position rather than attribution. We find that women in each position are systematically less likely than men to be named an author on any given article or patent for any given position that they occupy in the organization.

**Fig. 1:**
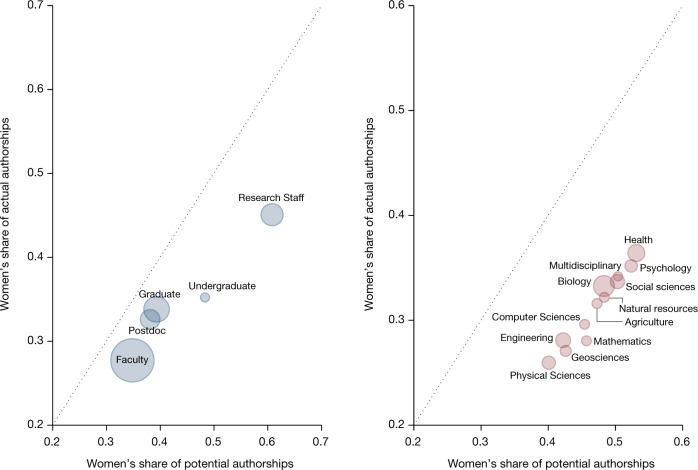
Women are less likely to be named authors on any given document in all fields and at all career stages. Graphs plot the probability that a potential author on a scientific document (article or patent) is a woman against the probability that an actual author is a woman. A potential author is defined as an employee in a laboratory between 2013 and 2016 from which an article or patent was published between 2014 and 2016. There are 17,929,271 potential article authorships and 3,203,831 potential patent inventorships in our sample. The markers in each panel are sized by the total number of actual authorships in the category. The diagonal represents parity in the gender composition of potential and actual authorships. Individual data on potential and actual authorships are shown in Supplementary Fig.  5. Left, disparity across job titles. Right, disparity across research fields. Observations are weighted by the inverse of the number of teams per employee times the inverse of the number of potential articles or patents per employee.

Figure 1 (and Supplementary Fig. 5) makes use of information in the data about each individual’s position in the organization—faculty, postdoc, graduate student, undergraduate student or research staff—as well as the research team’s field. Women occupy more junior career positions than men. The proportion of women in each position declines as the seniority of the position increases (Fig. 1, left). At the high extreme, 34.82% of faculty members are women; at the lower extreme, 60.81% of research staff are women.

However, Fig. 1 (left) also shows that the share of actual authorships for women is lower than what would be expected given their share of potential authorships in each career position. The difference between the share of potential authorships and actual authorships for women ranges from 15.72 pp for research staff (*P* = 0.0000; two-sided *t*-test; test value = −15.81; effect size = 15.72 pp) to 7.09 pp for faculty members (*P* = 0.0000; two-sided *t*-test; test value = −13.34; effect size = 7.09 pp) to 5.51 pp for postdocs (*P* = 0.0000; two-sided *t*-test; test value = −5.08; effect size = 5.51 pp; Extended Data Table 3). These gaps are clearly apparent as every marker Fig. 1 (left) is below the diagonal (also see Extended Data Table 3 and Supplementary Fig. 5).

A similar pattern is apparent when authorship is analysed by field (Fig. 1, right). For example, in biology, the share of actual authorships who are women is 15.02 pp lower than the share of women among potential authors (*P* = 0.0000; two-sided *t*-test; test value = −3,024; effect size: 15.02 pp; Extended Data Table 3). In physical science, the corresponding difference is 14.12 pp (*P* = 0.0000; two-sided *t*-test; test value = −25.44; effect size = 14.12 pp; Extended Data Table 3).  Note that the figure does not control for job title in disaggregating attribution by field, so fields with disproportionately more women and lower attribution rates may reflect the fact that there are more research staff.

**Fig. 2:**
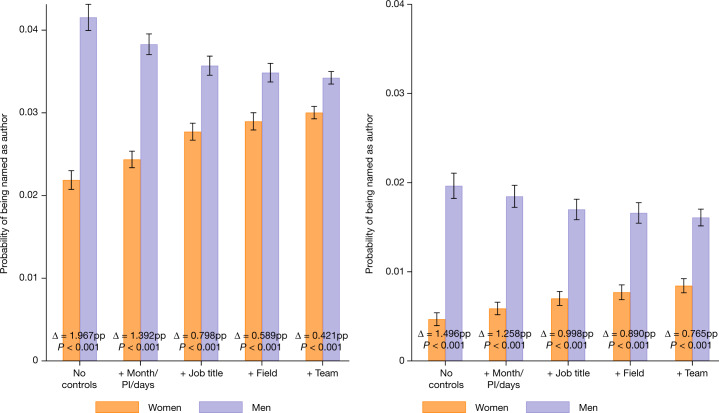
Women are still less likely to be named even when controls are included. Graphs show the probability that an individual in a team is an author on a given article (left) or patent (right) published by that team. Left, the likelihood of attribution on an article is estimated from 17,929,271 potential authorship observations. Right, the likelihood of attribution on a patent is estimated from 3,203,831 inventorship observations. The data associated with each bar are generated by predicting the dependent variable from ordinary least squares regressions of the likelihood of being named on gender and the indicated controls (reported in Extended Data Table 4). For the purpose of plotting probabilities and gender differences holding all else fixed (Δ), we hold all of the controls at their respective means. Because men have higher values than women on average on the controlled factors that increase the probability of attribution, the predicted probabilities for men decline and those for women increase as more controls are included. Controls, from left to right: (1) none; (2) whether a potential author is the PI of the team, the number of days worked on the team and publication date (calendar year × month); (3) job title of the potential author/inventor; (4) research field of the team; (5) individual indicator variables for each team (these team indicators subsume the fields indicator). The observations are weighted by the inverse of the number of teams per employee times the inverse of the number of potential articles or patents per employee.  Individual data on the probability of women or men being named on articles or patents are visualized in Supplementary Fig.  6. Error bars are centred on the mean and extend to the 95% confidence interval based on 1.96 × s.e. Standard errors are clustered by team and employee.

It is possible, of course, that the gender differences arise from compositional differences between women and men in the teams on which they work, fields, job titles or time allocated to particular projects. In particular, women might sort into teams with different propensities to publish or onto projects with different research questions. Figure 2 (and SI Figure S6) plots the estimated attribution rate for men and women on articles (left) and patents (right) as well as the differences (indicated by Δ). Using a series of regression models that control for these types of potential compositional differences, we estimated the attribution rate for men and women on articles and patents as well as the differences (indicated by Δ) (Fig. 2, Extended Data Table 4 and Supplementary Fig. 6). In these models, an indicator for being named is regressed on an indicator for gender as well as an increasingly expansive set of control variables (Extended Data Table 4). Column (1) includes no controls; column (2) adds publication date (calendar year × month), days worked on the team, and an indicator for the individual being a PI; column (3) adds job title indicators; column (4) adds field controls; and column (5) adds indicator variables for each team. Including these additional controls reduces, but does not eliminate, the disparity for women. Even in the fully specified model, which adds controls for each research team, women are 13.24% (*P* < 0.0001; two-sided *t*-test; test value = −6.3788; effect size = −0.4210 pp) less likely to be named on articles and 58.40% (*P*<0.0001; two-sided *t*-test; test value = −10.7746; effect size = −0.7652 pp) less likely to be named on patents.

The estimated regression-adjusted gender differences in attribution rates across job titles and fields, controlling for a wide variety of observable factors, are reported in Extended Data Tables 5 and 6. Notably, after including controls, the gender gap remains for all job titles except undergraduates. The gender gap similarly remains for 9 out of 13 fields for publications and 8 out of 13 fields for patents, after including controls.

**Fig. 3:**
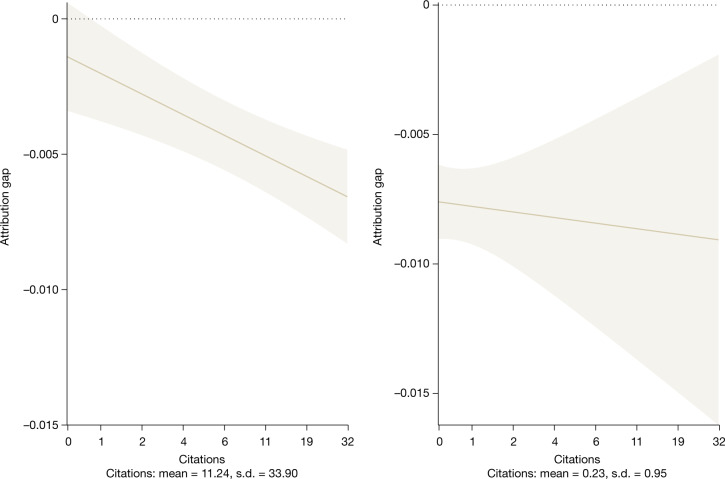
Women are much less likely to be named on high-impact articles. The probability that an individual in a team is an author on an article (left) or inventor on a patent (right) in relation to the number of citations that the document receives. Estimates were obtained from an ordinary least squares regression of the probability of being named with an indicator for gender against the log of total forward citations plus one (Extended Data Table 7). Left, the regression is estimated based on 17,929,271 potential article authorships. Right, the regression is estimated based on 3,203,831 potential patent inventorships. The observations are weighted by the inverse of the number of teams per employee times the inverse of the number of potential articles or patents per employee. Estimates include controls for publication date (calendar year × month), PI status, number of days worked on the team, job title and research team fixed effects. Each data point represents the estimated difference in the probability of a woman being named an author (left) or inventor (right) at each citation level. Error bars extend from the point estimate of the estimated marginal effect by ±1.96 × the standard error and show the 95% confidence interval of the marginal effect. Standard errors are clustered by team and employee.

The third measure reflects the fact that not all scientific documents are created equal. The omission of Franklin from the Crick and Watson paper was particularly egregious because of its high potential and ultimate scientific impact. The empirical implementation of the third measure is to attach forward citations to the articles and patents. Figure 3 shows that, when controlling for field, career position and team size, there is no significant difference between the likelihood of a woman being named relative to a man on an article with zero citations (*P* = 0.1725; two-sided *t*-test; test value = 1.3642; effect size = 0.1392 pp). However, for more highly cited articles women are less likely than men to be named. For example, on an article with 25 citations women are 19.9739% less likely to be named than men relative to the baseline (*P* < 0.0001; two-sided *t*-test; test value = −7.4982; effect size = 0.6352 pp; Extended Data Table 7).

## Attribution and survey data

Qualitative evidence about the reasons behind the lack of attribution can be obtained from surveys. Despite the well-known issues with selection bias, self-reporting and low response rates, survey data can be useful for triangulating against administrative data^20^. We designed a survey of authors who appeared on at least one article in the Web of Science^21^ after 2014 and who had a published and available e-mail address. We asked three core sets of questions of each individual to shed light on the findings from our analysis of administrative data (the full survey is reproduced in Supplementary Information, part 3).

To get a sense of how often scientists were not appropriately credited, we asked whether respondents had ever been excluded from a paper to which they had contributed. Out of 2,660 responses, there is a clear gender difference, with 42.95% of women and 37.81% of men having been excluded as an author (*P* = 0.0151; two-sided *t*-test; test value = −2.4327; difference = −0.0514). This gap is qualitatively similar to the gaps estimated using the administrative data, where men were almost twice as likely (21.17%) to be recognized as ever being an author or inventor as women (12.15%), and the attribution rate on potential authorships/inventorships for men was 4.23%, compared with 2.12% for women.

**Fig. 4:**
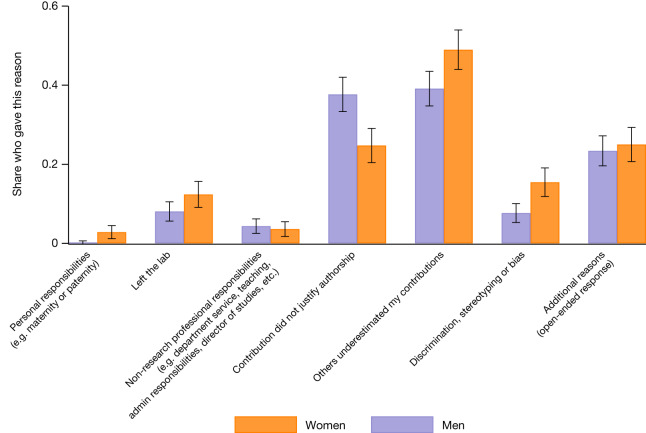
Women are more likely to report that their contributions were underestimated or that there was discrimination. A survey was sent to 28,000 scientists who had published in an academic journal listed in the Web of Science and who listed themselves with a public profile on the ORCID database. The bar chart shows the percentage of 871 men and women who provided answers to the survey question (Q2b): ‘What is the most likely reason that you were not listed as an author on that paper?’. Respondents were able to select more than one option, thus the total number of responses is higher than the number of respondents. The probability is computed as the arithmetic mean of the binary responses. Individual data on the reason an individual is not named are visualized in Supplementary Fig. 7. Error bars are centred on that mean and extend to the 95% confidence interval based on 1.96 × s.e.m. The difference in the probability of selecting ‘Contribution did not justify authorship’ between men and women is 0.1294 (*P* = 0.0000; two-sided *t*-test; test value = 4.1060). The difference in the probability of selecting ‘Others underestimated my contributions’ between men and women is −0.0984 (*P* = 0.0036; two-sided *t*-test; test value = −2.9218). The difference in the probability of selecting ‘Discrimination/stereotyping/bias’ between men and women is −0.0780 (*P* = 0.0003; two-sided *t*-test; test value = −3.6623). Additional *t*-tests of the differences in the probability of indicating a reason across men and women can be found in the text.

We also asked why respondents thought they were not credited: Fig. 4 (and Supplementary Fig. 7) summarizes the results for the 871 individuals who responded (483 men and 388 women). The most common reason was that scientific contributions were underestimated, and this was the case for far more women (48.97%) than men (39.13%) (*P* = 0.0036; two-sided *t*-test; test value = −2.9218; effect size = −0.0984). Although discrimination or bias was much less likely to be cited, women were twice as likely (15.46%) to cite this as a reason than men (7.67%) (*P* = 0.0003; two-sided *t*-test; test value = −3.6623; effect size = −0.0780). Men were more likely to say that their contributions did not warrant authorship (37.68% of men compared with 24.74% of women; *P* = 0.0000; two-sided *t*-test; test value = 4.1060; effect size = 0.1294). Differences in responsibilities (that is, a respondent indicated that they were not granted attribution for at least one of the following reasons: personal, non-research responsibilities and/or left the laboratory) appear to account for some of the attribution gap—17.53% of excluded women cited these reasons, compared with 12.63% of men (*P* = 0.0432; two-sided *t*-test; test value = −2.0244; effect size = −0.0490). Together, these estimates suggest that a large portion of the gender gap in attribution is owing to either discrimination or how contributions are perceived by collaborators, or both.

**Fig. 5:**
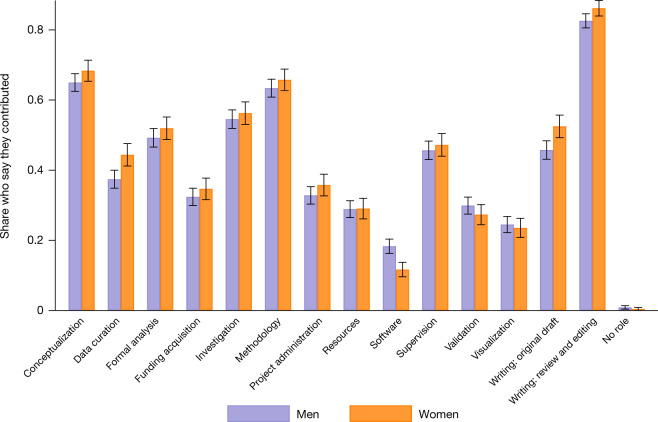
Women report making more contributions than men on authored papers. We sent a survey to 28,000 scientists who had published in academic journals listed in the Web of Science and who had a public profile in the ORCID database. Of these, 2,297 responded and completed the question (Q1a): ‘How did you contribute to the paper? Check all that apply.’ The graph shows the percentage of these respondents who selected each category. Probability was computed as the arithmetic mean of binary indicators representing whether the respondent selected each category. Each respondent was asked about a paper associated with them on Web of Science. Respondents were able to select more than one option, thus the total number of responses is therefore higher than the number of respondents. Individual data on the contribution by gender are visualized in Supplementary Fig.  8. Error bars are centred on the mean and extend to the 95% confidence interval based on 1.96 × s.e.m.

The same question—whether women with the same contribution as men are less likely to be credited—can be asked a different way: conditional on being credited, did women contribute more than men? Accordingly, we asked authors to indicate what they did to earn authorship on one of their most recent publications using the standardized contributions identified by Project Credit^22^. The results, reported in Fig. 5 and in Supplementary Fig. 7, are consistent: on average, women have to do more than men to be included as an author (2,297 individuals responded: 1,371 men and 926 women). A simple unweighted count of total contributions reported shows that women report a total 6.34 contributions on average compared with 6.11 for men (*P* = 0.0907; two-sided *t*-test; test value = −1.6925; effect size = −0.2376). Women report making significantly more contributions in conceptualization (64.99% of men versus 68.36% of women; *P* = 0.0937; two-sided *t*-test; test value = −1.6767; effect size = −0.0337), data curation (37.42% of men vs. 44.38% of women; p = 0.0008; two-sided *t*-test; test value = −3.3467; effect size of −.0697), writing the original draft (45.73% of men versus 52.48% of women; p = 0.0015; two-sided *t*-test; test value = −3.1813; effect size = −0.0675) and reviewing and editing (82.57% of men versus 86.18% of women; p = 0.0205; two-sided *t*-test; test value = −2.3178; effect size = −0.0361). The only category in which men reported a greater contribution was software (18.31% of men versus 11.67% of women; p = 0.0000; two-sided *t*-test; test value = 4.3174; effect size = 0.0664). There is no significant difference between men and women in either formal analysis (49.23% of men versus 51.94% of women; *P* = 0.2028; two-sided *t*-test; test value = −1.2740; effect size = −0.0271) or project administration (32.82% of men versus 35.75% of women; *P* = 0.1471; two-sided *t*-test; test value = −1.4504; effect size = −0.0292).

## Attribution and qualitative data

The third source of information was from the voices of scientists themselves. First, the survey permitted open-ended, written responses: 887 such responses were received. Three-hundred and thirty-eight respondents volunteered to be interviewed: 6 (4 women and 2 men) were selected for additional feedback. A number of cross-cutting themes emerged, in addition to expected differences across fields, research teams, countries and seniority.

The first was the importance of team structure and the role of voice: researchers felt that they had to advocate for themselves to be included, and if they are unaware or too unsure of themselves to speak up, they will miss out. As one woman respondent said, “I did not push to be listed as an author”. Another woman respondent noted that “Being a woman [means] that quite often you contribute in one way or another to science but unless you shout or make a strong point, our contributions are often underestimated.” Multiple respondents mentioned that a lack of voice could disproportionately affect women, minorities and foreign-born scientists. However, respondents also noted that speaking up could also backfire: “Senior authors shamed me in front of group for asking for recognition (trying not to be a female-doormat stereotype backfires pretty much every time I have tried...)”.

The second was a lack of clarity with respect to authorship rules, which reinforces organizational structure. Rules are often determined by senior researchers (who are disproportionately men), and are often governed by personal relationships and idiosyncratic preferences, which reportedly led to disagreements. In at least two interviews, and in many of the survey responses, the disagreements were extremely bitter. The open-ended responses included such statements as “Favoritism, narcissisms, power-play” (from a woman); “The team backstabbed me” (from a woman); “I […] found  this lack of credit from my PI to be childish and unprofessional” (from a man). Power imbalances were also frequently mentioned: for example, “Publications were used as reward and punishment. The department heads were on everything…[everything] was dependent on their decision on authorship. It was difficult to get away from them as it was a way to keep people tied to them” (from a woman).

Finally, interviewees and survey respondents were keenly aware of the importance of scientific output as a signal of scientific quality. They felt that being left off papers had important negative long-term consequences. Some felt that not getting credit had damaged their career: “My career would have been quite different with these two *Nature* papers” (from a woman); “Being left off papers for which I was one of the two main leads has greatly damaged my career as a researcher and my chance to get promotion, jobs, and grant funding. I am still an academic but in a teaching role” (from a woman); “Authorship is pivotal for career advancement, yet when trainees are excluded from authorship due to senior author decisions, there is no appeal or challenge process… most of my fellow academics (especially women, and most especially women of colour) have been harmed by faculty members who decide to award authorship to other laboratory members who did not do the work” (from a woman). Others were still traumatized by the experience: “It was a very tough experience and I am relieved it didn't happen earlier in my career because that would have been devastating” (from a woman); “I'm still very angry over this disgusting behavior” (from a woman); “[it was] one of the lowest points of my professional career” (from a woman).

## Discussion

The key finding of this work is that, regardless of the measure of scientific credit, and despite efforts to standardize credit^15^, women are much less likely than men to be credited with authorship. The results are robust to a variety of alternative specifications and sample restrictions described in detail in Supplementary Information, part 1, namely (1) differential accuracy of gender imputation for non-English and Asian names; (2) differential match quality because of name changes and frequency; (3) the definition of potential authors, including first and last authorship; (4) differences by type of research output and the timing of research output relative to employment; (5) heterogeneity across more disaggregated fields; (6) sample construction; (7) definition of time working in laboratories; (8) logistic model; and (9) combinations of robustness checks.

Thus, some of the well-documented ‘productivity gap’^1–5^ may not be a gap in the contribution of women to science at all, but rather a gap in how much their contributions are recognized. The associated qualitative work suggests that the standards determining scientific attribution are not well-known or understood by all parties and are frequently disregarded. The result appears to be that women are systematically disadvantaged. Although our focus here is on gender, these gaps were also reported in our survey for other marginalized groups.

The evidence presented here is consistent with the notion that gender differences in science may be self-reinforcing—that the fate experienced by Rosalind Franklin and others like her discouraged numerous potentially high-impact researchers from entering science^23^. The under-representation of women in faculty positions may be the result of early discouragement among junior researchers: women are less likely to be recognized for their contributions—especially on pivotal projects—and may consequently be less likely to advance in their careers. Longitudinal work on the progress of women’s careers^24^ could be furthered by studying these data, which could provide an empirical link between credit attribution, women’s career progression and discouragement of early-stage researchers.

There are also important caveats; each data source has its drawbacks. The administrative data are drawn from research-intensive universities; therefore, the research experiences described using the administrative data may not represent the research experiences for all teams and, to the extent that women may be under-represented in research-intensive universities, may not represent the experiences of all women. Similarly, although the survey data are drawn from a broader sample, they are drawn from a sample of authors, so they do not capture the experiences of those who have never been named as an author.

Much more can be done to unpack the findings in other dimensions, such as the mechanisms whereby credit for scientific work is allocated, other dimensions of identity, and richer (for example, non-binary and fluid) measures of gender. Although we made every effort to be aware of and to guard against confirmation bias^25,26^ by including a variety of robustness checks in the quantitative analysis, by working with survey methodologists to review the survey to ensure that the questions were not leading to a ‘desired’ answer^27^, and by developing an interview protocol that did not introduce any discussion of gender (Supplementary Information, part 3), we encourage other researchers to work with the code and data that are available at IRIS to extend our analyses. Indeed, the unique data infrastructure highlighted in this work can be, and is being, expanded^16^ by the addition of new universities and links to many different data sources. It can be used by many other researchers to allow more examination of the organization of science—ranging from rich and complex data on the dynamic longitudinal interactions on what is funded (grants), who is funded (PIs), and the characteristics of the individuals and research teams who are employed by those funds. It will also be possible in future work to examine the effect of policies instituted by the research institutions at which researchers work (at the department, campus and university level^28^) on the retention and productivity of scientists^29^, student placements and career trajectories^30–32^, as well as business startups^33^.

In sum, and beyond the results presented here, this paper serves as the introduction to a new and rich data infrastructure that is at least as rich as the bibliometrics data infrastructure that has served as the evidence basis for the study of the science of science^34^. The infrastructure, which is currently being used by more than 200 researchers can be, and has been, replicated in other countries^35^ and provides new insights into the organization of science.

## Methods

The Methods are divided into four parts. The first describes the data construction and variable operationalization used in the analysis of administrative data; the second describes the analysis of the administration data; the third describes the construction of the survey data; and the fourth describes the qualitative responses and interviews.

## Ethical approval

Institutional review board approval: University of Pennsylvania Institutional Review Board (IRB protocol no. 850522) approved the survey. University of Pennsylvania Institutional Review Board (IRB protocol no. 850522), Boston University Institutional Review Board (IRB protocol no. 6412X) the New York University Institutional Review Board (IRB protocol no. IRB-FY2022-6243) and the Ohio State University Institutional Review Board (IRB protocol 2022E0133) approved the follow-up interviews.

### Construction of administrative data

The analytical linked dataset, which consists of observations on 128,859 individuals employed on 9,778 research teams from 2013 to 2016 linked to 39,426 subsequent articles and 7,675 patents, is constructed from three sources: internal finance and human resources (FHR) administrative data from 20 universities and 57 colleges and campuses^36^, representing over 40% of total academic R&D spending in the United States, journal articles from the Web of Science and patent data derived from the universe of patents from the US Patent and Trademark Office.

### Finance and human resources data

The first source is derived from FHR data, called UMETRICS, on all personnel paid on sponsored research projects for 118 college campuses from 36 universities from 2001 to 2022 (the exact years covered vary by institution)^36^. A full list of participating institutions, which are primarily research-intensive, can be found at https://iris.isr.umich.edu/.

For each pay period, the FHR system at each university records the details of charges to each sponsored project, including for each person paid on each grant and reports the information to the Institute for Research on Innovation and Science^37^. These administrative data are different from the level-of-effort data that are submitted by PIs as part of their annual and final report to an agency in at least three ways. First, they represent actual payroll data, drawn from the FHR system every pay period, rather than the estimate provided by the PI or the team administrator once a year. An intensive hand-curated effort that compared the results from an early effort found that the FHR reports are more granular and comprehensive than the PI or team administrator reports^38,39^. For example, all personnel names (including co-PIs) are recorded in the FHR reports, but many names are not recorded in the former. Second, the UMETRICS data capture all sources of funding, and are much more comprehensive than data from a single agency. The UMETRICS data include federal funding sources as well as funding from philanthropic foundations, state and local governments, industry, and international organizations. Third, the data reflect actual expenditures in every accounting time period, not just funds that are obligated at the beginning of a grant. So if, as often happens, there is a no-cost extension, or more funds are spent earlier in the project, that spending and the work of the relevant team members is captured in the data. There are limitations. If personnel do not charge time to the grant, their effort is not captured in the data; we are unaware of any source that would capture unpaid work. If there are gender differences in unpaid research work, the analysis would not be able to capture such differences.

The analysis focuses on a subset of 57 college campuses from 20 universities, which consistently provided data for the period covering 2013–2016 (refer to pages 10, 11 and 23 of the UMETRICS summary documentation^36^; Supplementary information, part 2). This restriction ensures that employment spells are long enough to reasonably identify PIs and teams as well as to observe the scientific documents produced by those teams from 2014–2016. The full data include administrative-level information from 392,125 unique federal and non-federal awards, including 23,307,254 wage payments to 643,463 deidentified individuals^28^.

#### Research teams

The construction of research teams was informed by the work of Stephan^30^, who operationalized the concept of a research team to be a collection of scientists working jointly on projects with common funding and resources. The UMETRICS data are ideally suited to create measures of teams at scale using this definition, because the administrative data provide detailed information of all people charging time to each grant in each payroll period^31,32^.

The composition of each team is constructed as follows. The PI is at the centre of each team. The PIs in the data are identified by selecting faculty members who have been continuously paid on at least one research grant per year from 2013–2016 and whose associated wage payments always list faculty member as their job title. The PI-associated grants are identified if at least one wage payment was made to the PI during the sample period and shared evenly if they involve multiple PIs. Research centre grants, which are characterized as grants with 12 or more faculty members—the 99th percentile of the grants—were excluded. Based on the grants associated with the PI, we identify the set of graduate students, postdocs, research staff, undergraduates and non-PI faculty members who are paid on those grants. The set of scientists paid on the grants associated with the PI collectively make up the research team. This procedure yields a total of 9,778 teams, with 128,859 employees between 2013 and 2016.

The number of teams and potential authorships varies considerably across people in the sample. To ensure that our estimates are not dominated by people who are on many teams or on teams with many articles, we weight our data so that each person receives equal weight and for each person, each team receives equal weight. If ${N}_{{Teams},i}$ denotes the number of teams that person *i* is on and ${N}_{{PA},t}$ denotes the number of potential authorships (i.e., articles and/or patents) on team *t*, then the weight applied to person *i*’s potential authorships on team *t* is $\frac{1}{{N}_{{Teams},i}\cdot {N}_{{PA},t}}$. Thus, each person is weighted by the inverse of the number of teams on which he or she appears times the inverse number of potential authorships for that team. Each unique employee therefore has an overall weight of one in the sample. Our results, however, are robust to various alternative weightings.

#### Gender

Gender is algorithmically assigned using a combination of Ethnea^32,40^ and the Python Gender Guesser algorithms. Ethnea is first used to assign gender based on the first name and ethnicity (algorithmically assigned from the family name) of each employee. When the first name gives ambiguous results, the middle name is used. If gender is still ambiguous, Python’s Gender Guesser is applied to the individual’s first name, but not the middle name. Gender can be identified for 107,239 people (83.2% of sample), of whom 51,738 are women and 55,502 are men.

The accuracy of the imputation was tested against two sources of ground truth. The first source is self-reported, administrative data on gender for 12,867 faculty members from one institution participating in UMETRICS. The algorithm correctly predicts the self-reported gender in 93% of the cases: the precision is 93.35% for men and 92.51% for women. The second source is derived from a match of the UMETRICS data with the Survey of Earned Doctorates^41^. The Survey of Earned Doctorates, an annual survey (with a 93% response rate) of all doctorate graduates from US universities, directly asks respondent to report their gender. The precision of the algorithm was 97.29% for men and 94.06% for women. Robustness checks are reported in the Supplementary Information. We note the limitation that our gender construct does not allow for non-binary or fluid gender identities. Addressing non-binary and/or fluid gender identities is an important direction for future research.

#### Job titles

Job titles for each employee, which are also referred to in the text as positions, or roles, are constructed from the FHR records^42^. Some employees may hold different job titles on the same or different teams; in those instances, the title is equally weighted based on the number of days they were paid in each title within each team.

#### Scientific fields

The scientific field of each team is identified by using the title of all associated grants and comparing the grants with a pool of text that describes each scientific field using a wiki-labelling approach^17–19^. This approach is used to assign a likelihood score that a given grant award title belongs to a given research field category, as categorized by the NCSES Survey of Graduate Students and Postdoctorates in Science and Engineering. Each team’s field is estimated by taking the field of each grant and weighting the grant’s relative importance to the team’s portfolio by the direct expenditure of each grant over the analysis period.

#### Publications

Publications are drawn from the Web of Science database produced and maintained by Clarivate Analytics, which contains publication and citation information on approximately 69.3 million total articles from 1900 to 2018. The analysis focuses on articles published from 2014–2016 and linked to individuals observed in UMETRICS from 2013–2016, although we include some additional robustness checks on other year ranges and other publication types in the Supplementary Information.

#### Patents

Patents are drawn from the PatentsView visualization and analysis platform, which contains 6.8 million total patents dating from 1976 to 2018^43^. The analysis focuses on a subset of patents that have application dates between 2014 and 2016 and are linked to individuals observed in UMETRICS from 2013–2016. Additional robustness checks on other year ranges are include in the Supplementary Information.

#### Linked administrative records

The links between UMETRICS and authorship on articles and patents were generated by combining information on the individual and grants listed explicitly on the scientific documents as well as the implicit network structure of co-authorships and grant collaborations. In UMETRICS, the data include the individual’s name (including partial name in the case of hyphenated names), the institution and the grant number but, crucially, also other people on each grant. The same is the case in the publication and patent data. We identify all patents or articles associated with a given inventor or author by leveraging PatentsView’s algorithmically assigned inventor ID and the union of the Web of Science’s researcher ID and the ORCID when they are available. Key to our approach, these identity clusters enable us to link a given inventor or author’s full patent and publication history to an individual’s employee ID in UMETRICS such that we not only see those documents associated with a specific set of grants or a particular time period, but their entire patenting and publishing history over their career. The multi-step procedure, which uses data post 2000, is detailed in Ross et al.^44^. There are five steps. The first relies on an exact match of UMETRICS award numbers to either the award numbers cited in the government interest field in the patents or the award numbers cited in the acknowledgement section of the publication. The second step relies on name matches. It links inventors in Patentsview and authors in Web of Science to people paid on UMETRICS grants using a sequential process of exact and fuzzy matching, with matched names removed from the pool for subsequent rounds. Candidate matches are disqualified for mismatches on institutional affiliation and dissimilarity of text between awards and publications and patents. The third step relies on network matches. It uses exact and fuzzy name matching to find co-inventors (in Patentsview), co-authors (in Web of Science) and collaborators (in UMETRICS). Candidate matches are disqualified for mismatches on institutional affiliation and dissimilarity of text between awards and publications and patents. The fourth step links people by blocked affiliations. Affiliation names are matched by blocking on the UMETRICS university affiliation to the affiliations in PatentsView and Web of Science (using a hand-curated, disambiguated list of university names), and using the stepwise matching and validation processes described in the second step. As before, candidate matches are disqualified for mismatches on institutional affiliation and dissimilarity of text between awards and publications and patents. The fifth and final step relies on an approximate match of unmatched grants. It uses the pool of articles or patents associated with the identity clusters linked in steps 2–4 (namely, employees in UMETRICS linked with their associated inventor IDs and research ID or ORCID). The restriction that grant numbers on these documents are deterministically matched is loosened, and a fuzzy match is allowed between grants in UMETRICS and those unmatched in step 1 but associated with linked individuals.

#### Analytical sample

All publications and patents that acknowledge one of the team’s grants and/or has an author/inventor from the team are linked to the team. This results in a total of 47,101 scientific documents (39,426 articles and 7,675 patents) published between 2014–2016 which were linked to employees and teams observed in UMETRICS at any point in the previous year, that is, from 2013–2016. Summary information about the individuals and the teams is provided in Extended Data Table 1. Additional information about the differences between authors and non-authors in the sample as well as some basic descriptive information surrounding grant funding sources is provided in part 2 of the Supplementary Information.

The resulting linkages permit the calculation of the overall ever-author rate, which is 16.97% overall (12.15% for women and 21.17% for men) (Extended Data Table 2). The attribution rate is constructed by generating a pool of potential authorships as follows. All individuals with a faculty job title are considered eligible to be potential authors on all articles or patents produced by a team during the analysis period. All individuals with a non-faculty job title had to have been employed by the team in the year prior to the article of an article or application for a patent. We relax this time constraint for non-faculty job titles in the supplement, which generally increases the size of the gender gap reported in the main estimates.

The resulting analytical dataset consists of 21,133,102 potential authorship observations (17,929,271 on articles and 3,203,831 on patents) of which 367,231 were actual authorships. 43.8% of potential authorships were by women, whereas 31.8% of actual authorships were by women. If these numbers are converted to rates, the weighted attribution rate on scientific documents was 3.17%. The attribution rate for articles alone is 3.2% while it is 1.3% for patents (Extended Data Table 2). Although both of these attribution rates are relatively low, this is largely owing to the inclusion of undergraduate students and research staff in our sample as well as those observed working for short time periods. These employees are rarely observed in the actual authorships and result in a lower the overall attribution rate. The regression analyses reported in the subsequent sections control for both position and the number of days worked in the team; part 1 of the Supplementary Information provides results excluding undergraduates and research staff. The results are robust in each specification.

The third attribution measure—the impact of scientific articles and patents—is constructed by attaching forward citations (as of 2018) reported in the Web of Science and PatentsView datasets to the potential authorship sample. Because earlier documents in the sample (for example, those from 2014) have more time to receive citations than later documents (for example, those from 2016), we include publication date (calendar year × month) controls, as in our other models.

Effect sizes are calculated as the percentage point differences between the contrasted groups unless otherwise noted in the text.

### Empirical strategy

The empirical approach was to estimate linear regressions using a model of the form

1 $$P\left[\right.{{\rm{named}}}_{i,t,e,l}| \ldots ={\beta }_{0}+{\beta }_{1}{{\rm{woman}}}_{i,e}+{X}_{i,e}+{M}_{i,t}+{{\rm{O}}}_{i,e}+{{\rm{Team}}}_{i,l}+{\mu }_{i,t,e,l}$$

where *i* potential authorship observations are characterized by an employee *e* working on team *l* in the year prior to a document with a publication or application date *t* (calendar year × months). The primary variable of interest, ${{\rm{woman}}}_{i,e}$, is an indicator of whether a potential authorship was attributable to an employee who was a woman. Equation (1) is estimated on the sample of 17,929,271 potential authorships of journal articles, whereas the patent results are estimated on the sample of 3,203,831 potential inventorship.

A series of regressions was estimated. The first set (Extended Data Table 3) included controls, ${X}_{i,e}$, which sequentially include indicator variables for the publication or application month associated with a potential authorship or inventorship, the team’s PI, the number of days worked in the team, and an indicator of whether the individual’s gender was unknown. Idiosyncratic trends in the data are accounted for by including a series of ${M}_{i,t}$ calendar year × months and year fixed effects based on the date when article *i* was published or patent *i* applied for; an individual’s position in the team is accounted for through a series of ${O}_{i,e}$ position variables that capture the days that an individual worked in a particular position as a share of the total days worked on the research team. Differences across research teams are accounted by including a series of ${{\rm{Team}}}_{i,l}$ team fixed effects and we denote the disturbances in the data using ${\mu }_{i,t,e,l}$. The second set (Extended Data Table 5) re-estimated equation (1) with the same controls but by job title; the third set (Extended Data Table 6) re-estimated the same equation with the same controls by field. The final set (Extended Data Table 7) examined high-impact publications and patents.

### Survey design and collection

The survey was sent to individuals who had previously published in academic research journals identified through their public profiles on ORCID, a platform in which academic researchers post their educational credentials, work history and publication records. Information on the survey instrument, e-mail recruitment, and interview protocols is available in part 3 of the Supplementary Information.

The main database was the ORCID 2017 database, which includes the publicly viewable information from profiles shown on the ORCID website as they appeared in 2017: 897,264 profiles listed a complete name as well as educational credentials, work history information, or both.

E-mail addresses associated with the researchers of these profile were then derived from those e-mails listed on published and publicly available research articles available from the Web of Science. Web of Science also provides the associated e-mail addresses for 128,602 of the 897,264 ORCID profiles. Because the focus was on asking academic researchers about their experience with being named or not being named as co-authors on publications, the ORCID profiles were restricted to those that could be linked with a published academic paper in the Web of Science database between 2014 and 2018: 98,134 profiles fulfilled those criteria.

Finally, some individuals create multiple ORCID profiles and some e-mail addresses are recycled for multiple people over time. To avoid e-mailing the same individual multiple times, each e-mail had only one associated ORCID profile. After resolving duplicates, there were 98,022 unique ORCID profiles that matched our sample criteria.

Three studies were piloted before the main study. After imputing the gender of the individuals represented by the ORCID profiles using first names and the Ethnea database, 10,000 (imputed) ORCID profiles belonging to men and 10,000 (imputed) ORCID profiles belonging to women were randomly selected to receive the survey in addition to 6,500 profiles that had gender ambiguous names.

### Qualitative evidence

In addition to the open-ended text field in which researchers could record their experiences, the last question of the survey solicited researchers “to interview over Zoom regarding their experiences with the allocation of credit in research teams.” Respondents were told that if they were interested in talking about their experiences with the allocation of scientific credit on teams, they could enter their e-mail addresses to be contacted for a follow-up interview. A team of two authors (of both genders for three interviews, and of one gender for three interviews) of this paper interviewed six individuals for 30 min each. Four were women and two were men. Gender was never raised as an issue by the team but was raised by the interviewees. The detailed interview protocol is available in Supplementary Information, part 3.

### Reporting summary

Further information on research design is available in the Nature Research Reporting Summary linked to this paper.

## Online content

Any Nature Research reporting summaries, source data, extended data, supplementary information, acknowledgements, peer review information; details of author contributions and competing interests; and statements of data and code availability are available at 10.1038/s41586-022-04966-w.

### Supplementary information

Supplementary Information This file contains Supplementary Notes, Supplementary Figs. 1–9 and Supplementary Tables 1–12.

Reporting Summary

Supporting statistics for graphs.

## Figures and Tables

**Extended Data Table 1:**
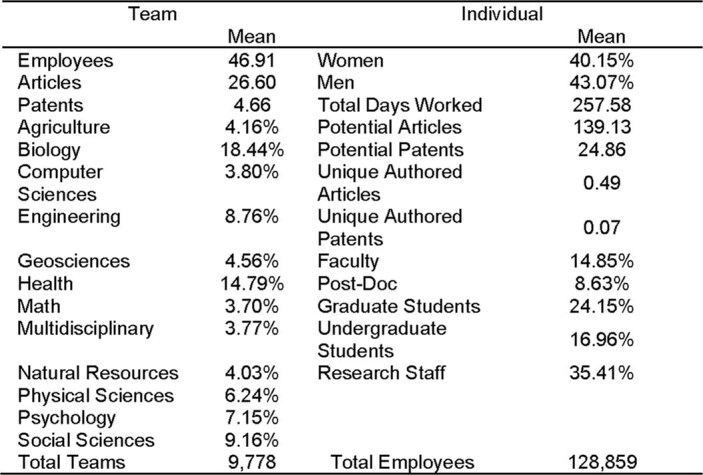
Team and Individual descriptive statistics

The table reports arithmetic means of the teams and the individuals who worked at least one day in any of the four years from 2013-16. Fields do not sum to 1 because fields are unassigned for about 11.5% of teams. Some employees hold different job titles over the timeframe; in those instances, they are first divided equally across titles within each team and then equally across teams. Shares of men and women do not sum to one because the gender of about 17% of the employees could not be algorithmically assigned.

**Extended Data Table 2:**
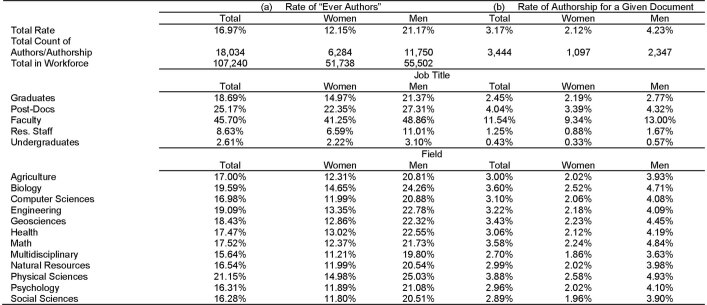
Attribution Rates by Job Title and Field

With the exception of two rows, “Total Count of Authors/Authorship” and “Total in Workforce”, the first three columns (a) summarize the share of “ever authors on a paper or ever inventors on a patent”. The numerator is individuals in each category who are ever named as an author on a publication or a patent. The denominator is the total number of individuals in that category. None of the total counts includes individuals whose gender could not be imputed. The second three columns (b) summarize the share of authorships: The denominator – the set of “potential authorships” – was created by associating all members of each team who were employed one year prior to the publication/application date of all associated articles/patents emanating from that team during the analysis period. Since some individuals, such as research staff, are on multiple teams, they are proportionately allocated across teams and papers using a set of analytical weights (see Methods Analytical Sample section for details). The numerator - attribution - was defined as “actual authorships” on those publications and patents (see Methods: Analytical Sample for details). The “Total Count of Authors/Authorship” row summarizes the total counts of “ever authors” in the first three columns (a) and the weighted total counts of actual authorships in the second three columns (b). The “Total in Workforce” row summarizes the total counts of individuals in our sample. Note that the “Total” column excludes those in our sample who we could not identify as either men or women.

**Extended Data Table 3:**
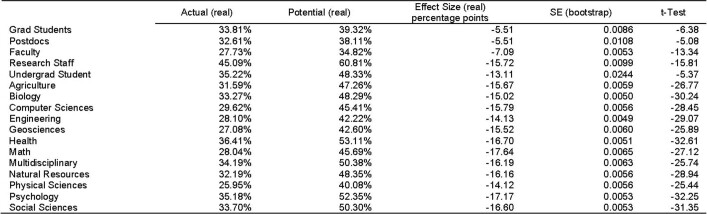
Gender Difference in Attribution Rate by Job Title and Field

The table summarizes the share of actual and potential authorships that are women. The first column shows the percentage of actual authorships who are women. The second column shows the share of potential authorships who are women. The third column provides the effect size, defined as the difference in percentage points between the share of actual authorships and the share of potential authorships who are women. The fourth column displays the estimated standard error for those differences based on the bootstrapping procedure described in the Methods: Analytical Sample section. The last column provides the two-sided t-test statistic for the effect size being equal to zero using the bootstrap estimated standard error. To generate standard errors, we drew samples of people with replacement and calculated the difference in the share of women among actual and potential authors.

**Extended Data Table 4:**
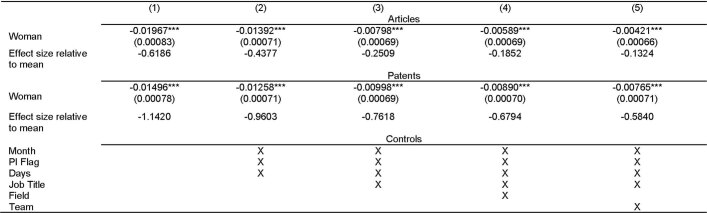
Gender differences in attribution

The sample consists of 17,929,271 potential article authorships and 3,203,831 potential patent inventorships. The top panel is estimated on the sample of potential article authorships and the bottom panel is estimated on the sample of potential patent inventorships. The dependent means are 3.18% and 1.31%, respectively. Specification (1) includes none of the control variables discussed above and estimates the gender gap to be 1.97 and 1.50 percentage points for articles and patents. Specifications (2-5) gradually introduce controls for days worked, PI status, publication month, job title, field, and team (which subsumes field). The observations are weighted by the inverse number of teams per employee times the inverse number of potential articles or patents per employee. Each coefficient is tested against the null hypothesis of being equal to 0 using a two-sided t-test. We do not adjust for multiple hypothesis testing. Standard errors are clustered by team and employee and are in parentheses. Statistical significance indicated by * p < 0.10, ** p < 0.05, *** p < 0.01.

**Extended Data Table 5:**
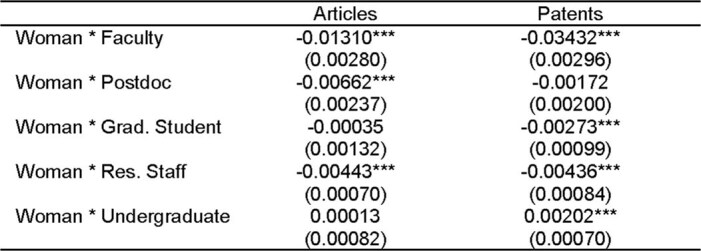
Gender differences in attribution by job title

Estimates based on a sample of 17,929,271 potential article authorships and 3,203,831 potential patent inventorships. The observations are weighted by the inverse number of teams per employee times the inverse number of potential articles or patents per employee. All estimates include controls for article/patent date (calendar year x month), PI status, days worked on the team, job title, and team fixed effects. Each coefficient is tested against the null hypothesis of being equal to 0 using a two-sided t-test. We do not adjust for multiple hypothesis testing. Standard errors are clustered by team and employee and are in parentheses. Statistical significance indicated by * p < 0.10, ** p < 0.05, *** p < 0.01.

**Extended Data Table 6:**
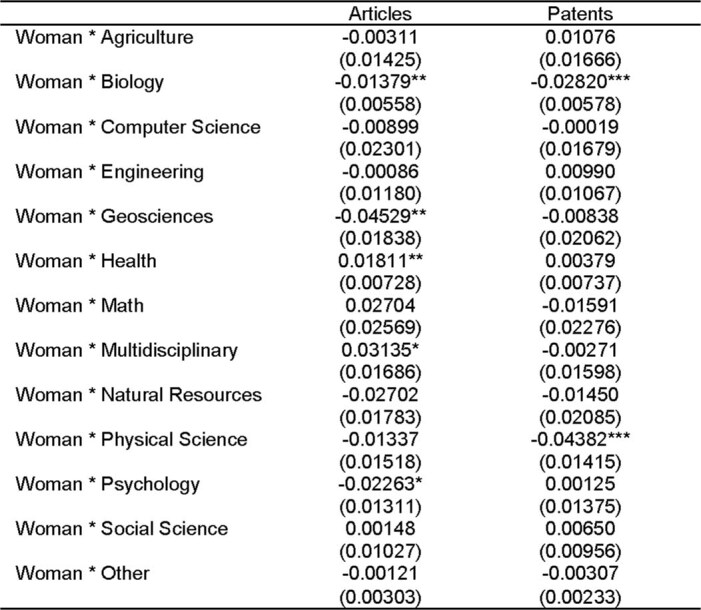
Gender differences in attribution by field

Estimates based on a sample of 17,929,271 potential article authorships and 3,203,831 potential patent inventorships. The observations are weighted by the inverse number of teams per employee times the inverse number of potential articles or patents per employee. All estimates include controls for article/patent date (calendar year x month), PI status, days worked in the team, job title, and team fixed effects. Each coefficient is tested against the null hypothesis of being equal to 0 using a two-sided t-test. We do not adjust for multiple hypothesis testing. Standard errors are clustered by team and employee and are in parentheses. Statistical significance indicated by * p < 0.10, ** p < 0.05, *** p < 0.01.

**Extended Data Table 7:**
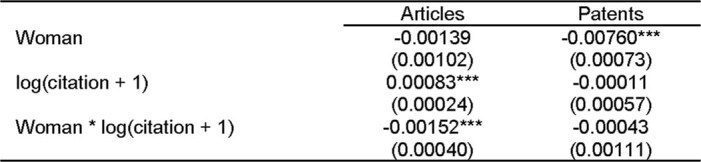
Gender differences in high impact attribution

Estimates based on a sample of 17,929,271 potential article authorships and 3,203,831 potential patent inventorships. The observations are weighted by the inverse number of teams per employee times the inverse number of potential articles or patents per employee. All estimates include controls for article/patent date (calendar year x month), PI status, days worked in the team, job title, and team fixed effects. Each coefficient is tested against the null hypothesis of being equal to 0 using a two-sided t-test. We do not adjust for multiple hypothesis testing. Standard errors are clustered by team and employee and are in parentheses. Statistical significance indicated by * p < 0.10, ** p < 0.05, *** p < 0.01.

**Extended Data Table 8:**
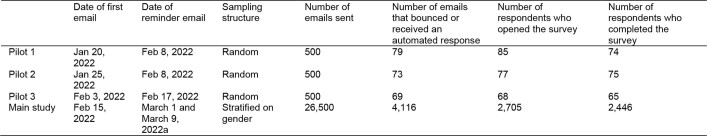
Survey Response Rates

This table describes the details of the three pilots and main study. The first column details the date of the first e-mail, while the second column details the date of the reminder e-mail, typically two weeks after the first e-mail. The main study had two reminder e-mails because on March 1st, an error in the Qualtrics survey system caused an abnormally high number of e-mails to bounce (~11,000). On March 9th, after the error had been addressed, we re-sent reminder e-mails to those respondents that had been missed due to the error. Column three describes the sampling strategy; random sampling was used for the pilots, but once we learned that there were far fewer women in the population than there were men, we adjusted to a gender-stratified sampling strategy in order to gain enough power for two-sided t-tests comparing responses from men and women. Specifically, 10,000 (imputed) ORCID profiles belonging to men and 10,000 (imputed) ORCID profiles belonging to women were randomly selected to receive the survey in addition to 6,500 profiles that had gender ambiguous names. Column 4 indicates the number of e-mails sent, while columns 5-7 document the response rate. A large fraction of emails either bounced or received an automated response.

**Extended Data Table 9:**
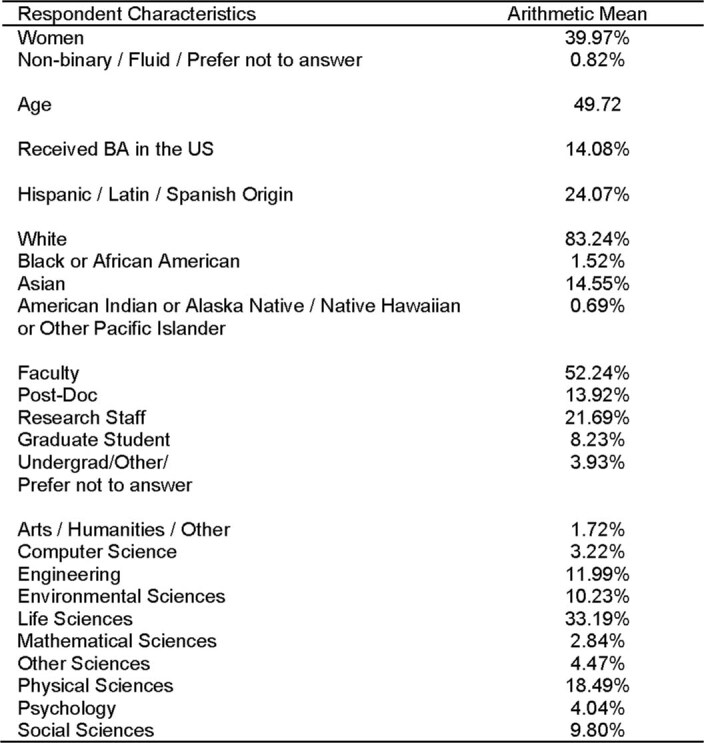
Characteristics of survey respondents

The table reports arithmetic means of the demographic characteristics of survey respondents.

## Data Availability

The datasets generated during and/or analysed during the current study are available at the Virtual Data Enclave repository at the Institute for Research on Innovation and Science at the University of Michigan. Access information is provided at https://iris.isr.umich.edu/research-data/access/. Patent data were obtained from PatentsView (https://patentsview.org/), which is publicly available. Web of Science data were obtained from CADRE at Indiana University (https://iuni.iu.edu/resources/datasets/cadre). The survey data are not available, per the University of Pennsylvania IRB protocols. Aggregate statistics from the survey data can be made available to researchers upon request, for replication purposes.

## Code availability

All the Stata code (version 17) and Python code (version 3.7.6) used is available in the Virtual Data Enclave at the University of Michigan. Access information is provided at https://iris.isr.umich.edu/research-data/access/.
