# Author Correction: Women are credited less in science than men

**DOI:** 10.1038/s41586-023-06571-x

**Published:** 2023-09-01

**Authors:** Matthew B. Ross, Britta M. Glennon, Raviv Murciano-Goroff, Enrico G. Berkes, Bruce A. Weinberg, Julia I. Lane

**Affiliations:** 1 Department of Economics and School of Public Policy and Urban Affairs, Northeastern University, Boston, MA USA; 2 Wharton School, University of Pennsylvania, Philadelphia, PA USA; 3 National Bureau of Economic Research, Cambridge, MA USA; 4 Questrom School of Business, Boston University, Boston, MA USA; 5 Department of Economics, Ohio State University, Columbus, OH USA; 6 Wagner School of Public Policy, New York University, New York, NY USA

**Keywords:** Economics, Sociology

Correction to: *Nature* 10.1038/s41586-022-04966-w Published online 22 June 2022

In the version of the article initially published, in the penultimate paragraph of the “Attribution and administrative data” section, the sentences now reading “Notably, after including controls, the gender gap remains for all job titles except undergraduates. The gender gap similarly remains for 9 out of 13 fields for publications and 8 out of 13 fields for patents, after including controls” have been corrected from “Notably, after including controls, the gender gap is significant for all job titles except undergraduates. The gender gap is similarly significant for 9 out of 13 fields for publications and 5 out of 13 fields for patents, after including controls”. In the “Ethical approval” section, under “Analytical sample”, “weighted” was added to the sentence now reading “If these numbers are converted to rates, the weighted attribution rate on scientific documents was 3.17%”. These changes have been made to the HTML and PDF versions of the article.

