# Supplementary material for: Women are credited less in science than men
Source: Nature. 2022 Jun 22;608(7921):135–45. doi: 10.1038/s41586-022-04966-w (PMC9352587; doi:10.1038/s41586-022-04966-w)
Supplement: Supplementary file 1 — This file contains Supplementary Notes, Supplementary Figs. 1–9 and Supplementary Tables 1–12. [file 41586_2022_4966_MOESM1_ESM.docx]

**Supplementary Information for “Women are Credited Less in Science than Their Male Counterparts”**

Contents

[Part 1: Robustness checks 2](#_Toc105762633)

[(i). Accuracy of gender imputation for Non-English and Asian names 2](#_Toc105762634)

[(ii). Match quality given name changes 4](#_Toc105762635)

[*Evidence on name changes* 4](#_Toc105762636)

[*Gender Bias in Web of Science Output* 6](#_Toc105762637)

[*Differences in Name Frequencies* 6](#_Toc105762638)

[(iii). Definition of potential authors 7](#_Toc105762639)

[*First and last authorship* 8](#_Toc105762640)

[(iv) Different types and timing of research output 8](#_Toc105762641)

[*Timing of output relative to employment* 9](#_Toc105762642)

[(v). Field Heterogeneity 10](#_Toc105762643)

[(vi). Sample Construction 11](#_Toc105762644)

[(vii). Research experience controls 12](#_Toc105762645)

[(vii). Logistic model 13](#_Toc105762646)

[Part 2: Descriptive Statistics 15](#_Toc105762647)

[(i) Information about the university sample 15](#_Toc105762648)

[(ii) Characteristics of Matched Authors/Inventors 15](#_Toc105762649)

[(iii) Funding Sources 16](#_Toc105762650)

[(iv) Counts of Authors, Workforce, and Authorship 16](#_Toc105762651)

[Part 3: Survey and Interview Details 18](#_Toc105762652)

[(i) Email Recruitment Script 18](#_Toc105762653)

[(ii) Survey 19](#_Toc105762654)

[(iii) Interview Protocol 26](#_Toc105762655)

[Part 4: Complementary View of Figures in Main Text 28](#_Toc105762656)

[References 32](#_Toc105762657)

# Part 1: Robustness checks

## (i). Accuracy of gender imputation for Non-English and Asian names

Although the gender imputation is quite precise, it is lower for non-English names, and especially for Asian names. The degree and effect of any resultant bias can be tested in three ways: (1) testing the robustness of the main results on a subsample that excludes scientists with an Asian name, (2) estimating the main results on a subsample that only includes scientists with an English name, and (3) implementing a Monte Carlo simulation that randomly assigns gender to those with an algorithmically unassigned gender. Table S1 provides the results for the first two robustness checks, where column 1 is the baseline, and corresponds to column 5 of Extended Data Table 4. The second and third columns show the first two robustness checks. The results for these robustness tests are qualitatively similar to the main results in terms of magnitude and the gender gap is estimated with a high degree of statistical precision.

Table S1: Gender differences in attribution

|  | Baseline | Excluding Asian Names | Only English Names |
| --- | --- | --- | --- |
|  |  | Articles |  |
| Woman | -0.00421^***^ | -0.00490^***^ | -0.00380^***^ |
|  | (0.00066) | (0.00068) | (0.00092) |
| N= | 17929271 | 14952969 | 7457304 |
| Dep. Mean | 0.032 | 0.030 | 0.030 |
|  |  | Patents |  |
| Woman | -0.00765^***^ | -0.00708^***^ | -0.00819^***^ |
|  | (0.00071) | (0.00070) | (0.00097) |
| N= | 3203831 | 2491000 | 1187322 |
| Dep. Mean | 0.013 | 0.011 | 0.011 |
| Calendar year x month | X | X | X |
| PI Flag | X | X | X |
| Days | X | X | X |
| Job Title | X | X | X |
| Field | X | X | X |
| Team | X | X | X |

Note: The observations are weighted by the inverse number of teams per employee times the inverse number of potential articles or patents per employee. Controls for article/paper calendar year x month, days worked in the team, a PI flag, job title, and an indicator for each team are included. Each coefficient is tested against the null hypothesis of being equal to 0 using a two-sided t-test. We do not adjust for multiple hypothesis testing. Standard errors are clustered by team and employee and are in parentheses. The statistical significance of each test is indicated by * p < 0.10, ** p < 0.05, *** p < 0.01.

The Monte Carlo simulation re-estimates the baseline model with full controls but randomly assigns a gender to the scientists with names that were not assigned through the imputation. ^[[1]](#footnote-1)^ This exercise is repeated 1,000 times to obtain a distribution of possible alternative point estimates corresponding with column 5 of Extended Data Table 4. These distributions provide a range of possible estimates that we could possibly get if these individuals had been assigned a gender. Figure S1 reports the distribution of estimates obtained through the simulation. The core findings from the main text remain unchanged: the gender gap never approaches zero or loses statistical significance.^[[2]](#footnote-2)^

Figure S1: Monte Carlo simulation results


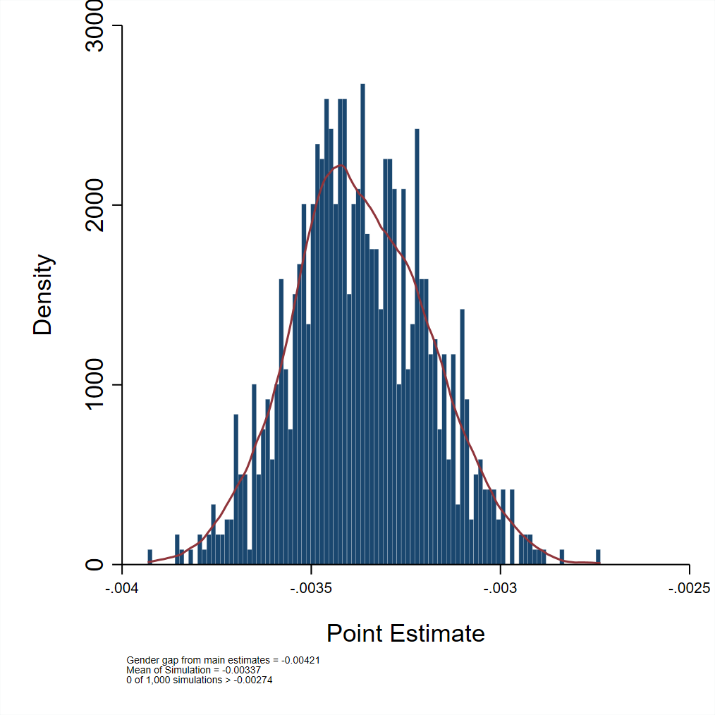

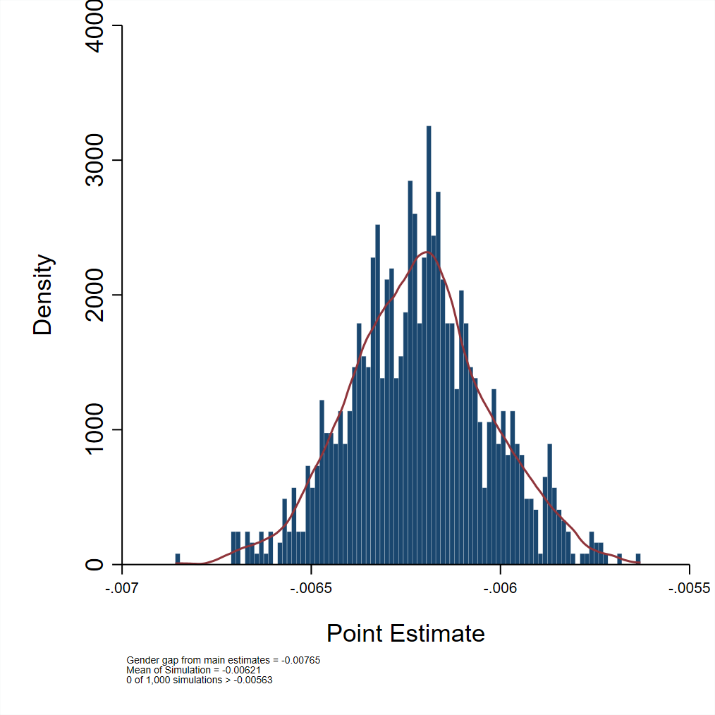


(a) (b)

Note: The figure shows the gender gap coefficient for articles (panel a) and patents (panel b) in percentage point terms from the Monte Carlo simulation of estimates on patents and articles.

A separate check of the robustness of the gender imputation is based on a manual analysis of scientific CVs. The team randomly drew 100 ORCIDs. Of those 100, the team was able to find 60 CVs. It was possible to determine self-reported gender for 59 individuals.^[[3]](#footnote-3)^ Of the 35 CVs belong to perceived men, gender was correctly imputed for 30 of them (86%); there were no incorrect imputations. Of the 24 CVs belonging to perceived women CVs, 20 (83%) could be imputed. Of those, 95% were correctly imputed to be women while one (5%) was incorrectly imputed to be a man. Overall, gender was imputed for 85% and of those with imputed gender, 98% were correctly imputed.

## (ii). Match quality given name changes

This section examines three possible systemic reasons for a differential match quality between the names of men and women from the employment records to the article data. The first is systematic differences in match rates due to scientists who are women changing their last name. The second concern is that there is a gender bias in the coverage of *Web of Science* articles. The third is differences in name frequency between women and men. None of these possible sources of bias appear to have had a substantive impact on the main results.

### Evidence on name changes

There is an extensive literature on marital name changes. Goldin and Shim provide an overview of that literature, and also do their own empirical analysis based on data from New York Times wedding announcements, Harvard alumni surveys, and Massachusetts birth records[1]. They identify a number of correlates of name “keepers”. One is age at marriage: older brides are much more likely to retain their names. Another is education – the fraction of college graduates who keep their name is about one in five; the probability increases by 15-29% if the bride has a PhD and about 20% more if the groom has a PhD. A third is religion – those who marry in a religious ceremony are less likely to be “keepers”. Rates are highest for women who have already “made a name” for themselves in a profession to “protect the value of their contacts, articles and professional goodwill”. In summary,

“.. the predicted probability she would change her name after marriage if she had a Ph.D., married a Ph.D. ten years after graduation and had no children was 0.069; the actual figure in the data is 0.059. The quantitatively most important components in explaining these large differences are those concerning whether the woman “made a name” for herself before marriage.” (Goldin and Schim, p.159)

Although we are not aware of a detailed analysis of the propensity of women scientists to change their name, Mary Frank Fox’s work[2] suggests that women academic scientists (who typically have PhDs) are very likely to marry other scientists (who have PhDs) and marry later. They are also likely, if they are in research teams, to have “made a name” for themselves. We would thus expect the rate of name changing in the analytical sample to be quite low.

Two data sources were used to examine the empirical rate of name changes for publishing scientists. The first was to compare the change in the number of last names used by researchers who are men and researchers who are women across the research articles recorded in the *Web of Science* over the course of their careers. The *Web of Science* database records the names of the authors for each article, as well, if provided, their Open Researcher and Contributor ID ([ORCID](https://orcid.org/)).^[[4]](#footnote-4)^ Gender was assigned to the researchers based on their names using the same gender algorithm procedure described in the Methods section. The mean number of distinct last names per ORCID was then calculated for researchers who are men and researchers who are women, stratified by the number of articles to control for gender differences in career lengths and article rates. The results, presented in Table S2, suggest that the difference in name changes is quite small. For example, men with two articles had an average of 1.0015 last names on those articles while women with two articles had an average of 1.0021 (or 0.06pp) more last names. The difference in means is small and not statistically significant for researchers with 2, 3, 4, or 5-10 articles. There is a small but statistically significant differences in the mean number of last names for authors with more than 10 articles.

Table S2: The number of last names associated with a given ORCID ID

| Article | Mean Number of Distinct Last Names | | |
| --- | --- | --- | --- |
| Number of. Articles | Men | Women | Percentage Point Difference |
| 2 | 1.0015 | 1.0021 | 0.06 |
| 3 | 1.0031 | 1.003 | -0.01 |
| 4 | 1.0029 | 1.005 | 0.21 |
| 5-10 | 1.0039 | 1.0058 | 0.19 |
| 10+ | 1.0038 | 1.007 | 0.32 |

The second approach was to examine the rate of name changes among life scientists, by using the rich information in the NIH grant database identified by Core Project numbers awarded between 2000 and 2018. This database records a unique PI ID, constant across grants, as well as the reported last name, which can vary across grants. It is thus possible to track the number of last names per Principal Investigator (PI) ID on NIH grants.

The same approach was used as in the first analysis. Gender was assigned to the researchers based on their names using the same gender algorithm procedure described in the Methods section. As before, the mean number of distinct last names per PI ID was then calculated for men and women, stratified by the number of articles to control for gender differences in career lengths and article rates. Table S3 reports the average number of distinct last names per NIH PI ID for men and women. Women with two NIH grants are only slightly more likely to have changed their last name between receiving the grants than were men, and the order of magnitude of the name change difference is much less than the attribution differences reported in the main text.

Table S3: The number of last names associated with a given NIH PI ID

|  | Mean Number of Distinct Last Names | | |
| --- | --- | --- | --- |
| Number of Grants | Men | Women | Percentage Point Difference |
| 2 | 1.0012 | 1.0155 | 1.43 |
| 3 | 1.0018 | 1.0162 | 1.44 |
| 4 | 1.0019 | 1.0152 | 1.33 |
| 5-10 | 1.003 | 1.0101 | 0.71 |
| 10+ | 1.0012 | 1.0155 | 1.43 |

### Gender Bias in Web of Science Output

A second related concern is that the *Web of Science* might not be equally representative of the output of men and women, because women might be more likely to publish in different outlets. A more detailed analysis of the random sample of 100 CVs described above, revealed that article information was available on 42 of the CVs. A comparison of the number of articles reported by faculty on those 42 CVs with the articles reported for the same individuals in the *Web of Science* revealed that the women had slightly more articles in the *Web of Science* than men, although the difference was not statistically significant. The correlation in the number of articles between the CVs and *Web of Science* data was .66 for men and .73 for women. These results ameliorate the concern that *Web of Science* is differentially representative by gender.

### Differences in Name Frequencies

Uncommon names are inherently easier to link. If women’s names are less common than men’s names, there could be systematic gender bias in the linkage results. This potential issue was assessed by creating name frequencies based on unique authors identified by either an ORCID or a WoS Researcher ID, and characterizing names as rare or common based on whether their associated frequency lies below or above the mean. The indicators for rare and common names are linked back to UMETRICS. The preferred empirical specifications are estimated for the sample of individuals with rare and common last names as well as for the combination of their first and last name.^[[5]](#footnote-5)^ The estimates remain strongly negative and statistically significant for both subgroups. (Table S4).

Table S4. Gender Differences in Articles by Frequency of Name

|  | Baseline | Rare  (Last Name) | Common  (Last Name | Rare  (First + Last Name) | Common  (First + Last Name) |
| --- | --- | --- | --- | --- | --- |
| Woman | -0.02798^***^ | -0.03737^***^ | -0.01937^***^ | -0.02444^***^ | -0.02184 |
|  | (0.00571) | (0.00996) | (0.00710) | (0.00592) | (0.02046) |
| N= | 1635666 | 501011 | 1134635 | 1386877 | 248769 |
| Dep. Mean | 0.230 | 0.237 | 0.228 | 0.232 | 0.220 |
| Calendar year x month | X | X | X | X | X |
| PI Flag | X | X | X | X | X |
| Days | X | X | X | X | X |
| Job Title | X | X | X | X | X |
| Field | X | X | X | X | X |
| Team | X | X | X | X | X |

Note: The observations are weighted by the inverse number of teams per employee times the inverse number of potential articles and patents per employee. Controls for article/patent calendar year x month, days worked in the team, a PI flag, job title, and an indicator for each team are included. Each coefficient is tested against the null hypothesis of being equal to 0 using a two-sided t-test. We do not adjust for multiple hypothesis testing. Standard errors are clustered by team and employee and are in parentheses. The statistical significance of each test is indicated by * p < 0.10, ** p < 0.05, *** p < 0.01.

It is worth noting that, while uncommon names are inherently easier to link between WoS and UMETRICS, the matching procedure uses considerably more information than just the name. The initial round of name matching uses grant acknowledgments on patents and grants to identify high probability matches. Once these matches have been made, additional matches are identified by crawling through the network of collaborators on grants and co-authors on articles / co-inventors on patents. At each stage, all matches are validated by comparing the text on an author’s associated grants and patents.

## (iii). Definition of potential authors

It is possible that defining potential authors to include all members of a research team is too expansive. The robustness of the main results was tested by re-estimating the regressions on two more subsamples: (1) a sample that excludes job titles with a low overall attribution rate, that is undergraduate students and research staff; and (2) a sample of those from UMETRICS who appear on a scientific document at least once from 2000-19 and that we have linked to an ORCID or WoS Researcher ID for the publication sample and a PatentsView Inventor ID for the patent sample.^[[6]](#footnote-6)^

The main results corresponding to these more restrictive subsamples are shown in Table S5. Column 1 reports the baseline result from column 5 of Extended Data Table 4, column 2 reports results for the sample excluding undergraduates and research staff, and column 3 restricts the sample to only those individuals who have been matched to an ORCID and WoS Researcher ID or PatentsView Inventor ID. These robustness tests result in a statistically precise estimate that is consistent in sign relative to the main results and broadly similar in magnitude in relation to the dependent means.

.

Table S5: Gender differences in attribution by different samples

|  | Baseline | Excluding Undergraduates and Research Staff | Only Matched Authors |
| --- | --- | --- | --- |
| Articles | | | |
| Woman | -0.00421^***^ | -0.00577^***^ | -0.02798^***^ |
|  | (0.00066) | (0.00130) | (0.00571) |
| N= | 17929271 | 9775106 | 1635666 |
| Dep. Mean | 0.032 | 0.055 | 0.230 |
| Patents | | | |
| Woman | -0.00765^***^ | -0.01329^***^ | -0.06041^***^ |
|  | (0.00071) | (0.00135) | (0.01336) |
| N= | 3203831 | 1942550 | 442350 |
| Dep. Mean | 0.013 | 0.023 | 0.245 |
| Calendar year x month | X | X | X |
| PI Flag | X | X | X |
| Days | X | X | X |
| Job Title | X | X | X |
| Field | X | X | X |
| Team | X | X | X |

Note: The observations are weighted by the inverse number of teams per employee times the inverse number of potential articles per employee. Controls for article calendar year x month, days worked in the team, a PI flag, job title, and an indicator for each team are included. Each coefficient is tested against the null hypothesis of being equal to 0 using a two-sided t-test. We do not adjust for multiple hypothesis testing. Standard errors are clustered by team and employee and are in parentheses. The statistical significance of each test is indicated by * p < 0.10, ** p < 0.05, *** p < 0.01.

### First and last authorship

The main text focuses on inclusion as an author regardless of position. This section reports the robustness of results in Table S6 when authorship is defined as either first or last authorship only. The gender gap persists, but the estimates are less negative than the baseline estimates shown in the first column (replicated from column 5 of Extended Data Table 4). This result was uncertain a priori: while first and last authorship is an even rarer event that authorship overall, and makes a smaller effect more likely, the qualitative results suggest it is harder to drop someone who played a leading role and whose author position may have been established up front, making a less negative effect more likely.

Table S6. Gender differences in articles by author position

|  | | Baseline | (1) | (2) | (3) | (4) | | (5) |
| --- | --- | --- | --- | --- | --- | --- | --- | --- |
|  | Articles, First Author | | | | | | | |
| Woman | | -0.00421*** | -0.00382^***^ | -0.00262^***^ | -0.00041 | | -0.00016 | -0.00014 |
|  | | (0.00066) | (0.00039) | (0.00038) | (0.00038) | | (0.00038) | (0.00036) |
| N= | | 17929271 | 17929271 | 17929271 | 17929271 | | 17929271 | 17929271 |
| Dep. Mean | | 0.032 | 0.010 | 0.010 | 0.010 | | 0.010 | 0.010 |
|  | Articles, Last Author | | | | | | | |
| Woman | | -0.00421*** | -0.00652^***^ | -0.00388^***^ | -0.00242^***^ | | -0.00142^***^ | -0.00098^***^ |
|  | | (0.00066) | (0.00036) | (0.00031) | (0.00031) | | (0.00032) | (0.00032) |
| N= | | 17929271 | 17929271 | 17929271 | 17929271 | | 17929271 | 17929271 |
| Dep. Mean | | 0.013 | 0.007 | 0.007 | 0.007 | | 0.007 | 0.007 |
| Calendar year x month | | X | - | X | X | | X | X |
| PI Flag | | X | - | X | X | | X | X |
| Days | | X | - | X | X | | X | X |
| Job Title | | X | - | - | X | | X | X |
| Field | | X | - | - | - | | X | X |
| Team | | X | - | - | - | | - | X |

Note: The observations are weighted by the inverse number of teams per employee times the inverse number of potential articles or patents per employee. Controls for article/patent calendar year x month, days worked in the team, a PI flag, job title, and an indicator for each team are included. Each coefficient is tested against the null hypothesis of being equal to 0 using a two-sided t-test. We do not adjust for multiple hypothesis testing. Standard errors are clustered by team and employee and are in parentheses. The statistical significance of each test is indicated by * p < 0.10, ** p < 0.05, *** p < 0.01.

## (iv) Different types and timing of research output

In the main analysis, articles are restricted to those classified by WoS as journal articles. However, research has shown that the contributions of women and minorities are often overlooked due to a narrow focus on journal articles[3]. It is possible that women are more likely to publish in slow science (books) and fast science (conference proceedings) than in journal articles, and hence that the gender gap identified in the main text reflects gender differences in the type of output.

The results are robust to an expansion of the definition of articles to include other types of output beyond journal articles – books, conference proceedings, and all other documents (such as data papers, reports, and reviews).^[[7]](#footnote-7)^ Table S7 reports the results, where each column corresponds to the samples described. The sign on the coefficient for “woman” remains negative for every sample, indicating the magnitude of the gender gap in attribution is consistent across all output types. Of course, other types of output such as managerial tasks, mentoring graduate students, or teaching are not captured, and may well differ by gender.

Table S7: Gender differences in attribution by type of output

|  | (1) | (2) | (3) | (4) |
| --- | --- | --- | --- | --- |
|  | Baseline | Books | Conference Proceedings | All Other  Documents |
| Woman | -0.00421^***^ | -0.00467^*^ | -0.00328^***^ | -0.00476^***^ |
|  | (0.00066) | (0.00250) | (0.00107) | (0.00079) |
| N= | 17929271 | 20746 | 286425 | 2015085 |
| Dep. Mean | 0.032 | 0.007 | 0.011 | 0.019 |
| Calendar year x month | X | X | X | X |
| PI Flag | X | X | X | X |
| Days | X | X | X | X |
| Job Title | X | X | X | X |
| Field | X | X | X | X |
| Team | X | X | X | X |

Note: The observations are weighted by the inverse number of teams per employee times the inverse number of potential articles or patents per employee. Controls for article/patent calendar year x month, days worked in the team, a PI flag, job title, and an indicator for each team are included. Each coefficient is tested against the null hypothesis of being equal to 0 using a two-sided t-test. We do not adjust for multiple hypothesis testing. Standard errors are clustered by team and employee and are in parentheses. The statistical significance of each test is indicated by * p < 0.10, ** p < 0.05, *** p < 0.01.

### Timing of output relative to employment

The main results identify researchers employed on projects in 2013-2016 and their articles and patents in the next year. In other words, only those employed on a team in the year prior to an article/patent’s publication date are considered as eligible authors. However, it is possible that women might be excluded from articles or patents published over a longer period. The baseline regression (column 5 of Extended Data Table 4) is accordingly re-estimated for that subset of individuals who were last observed in 2013 (i.e., who left the team in that year) and include the articles and patents produced by their team in the subsequent years, up to 2018. Each year is considered separately, and the resulting estimates are reported in Table S8 for articles and patents. The gender gap remains statistically significant and broadly in line with the baseline estimates. The only insignificant estimate is for articles in 2018: the ties to the team are likely to have weakened substantially by then.

Table S8. Gender difference in attribution by year for authors/inventors that exit the sample in 2013

|  | (1) | (2) | (3) | (4) | (5) |
| --- | --- | --- | --- | --- | --- |
|  | 2014 | 2015 | 2016 | 2017 | 2018 |
| Articles | | | | | |
| Woman | -0.00566^***^ | -0.00410^***^ | -0.00388^***^ | -0.00457^***^ | -0.00172 |
|  | (0.00096) | (0.00083) | (0.00073) | (0.00075) | (0.00243) |
| N= | 5783233 | 6608340 | 7081463 | 6332569 | 5863 |
| Dep. Mean | 0.034 | 0.031 | 0.029 | 0.028 | 0.002 |
| Patents | | | | | |
| Woman | -0.00893^***^ | -0.00763^***^ | -0.00623^***^ | -0.00676^***^ | -0.00689^***^ |
|  | (0.00097) | (0.00083) | (0.00070) | (0.00070) | (0.00073) |
| N= | 1,084,175 | 1,196,418 | 1,436,690 | 1,568,855 | 1,456,466 |
| Dep. Mean | 0.014 | 0.012 | 0.011 | 0.011 | 0.011 |
| Calendar year x month | X | X | X | X | X |
| PI Flag | X | X | X | X | X |
| Days | X | X | X | X | X |
| Job Title | X | X | X | X | X |
| Field | X | X | X | X | X |
| Team | X | X | X | X | X |

Note: The observations are weighted by the inverse number of teams per employee times the inverse number of potential articles or patents per employee. Controls for article/patent calendar year x month, days worked in the team, a PI flag, job title, and an indicator for each team are included. Each coefficient is tested against the null hypothesis of being equal to 0 using a two-sided t-test. We do not adjust for multiple hypothesis testing. Standard errors are clustered by team and employee and are in parentheses. The statistical significance of each test is indicated by * p < 0.10, ** p < 0.05, *** p < 0.01.

## (v). Field Heterogeneity

Attribution is likely to be at least partly driven by different norms across fields, as evidenced by the differences in Figure 1 and Extended Data Table 4. Field controls (or team fixed effects which subsume these controls) were included in some specifications (including our preferred specification).

However, the primary specifications reported in the main text use relatively highly aggregated fields (12), and one might expect significant differences within a broad field like social sciences. To test whether the results are robust when controls for an even more disaggregated set of fields are used, the field definitions were expanded to the 91 detailed GSS categories used for the Survey of Graduate Students and Postdoctorates in Science and Engineering. As in the baseline analysis, we use a “Wiki-labeling” approach to generate the field concordance[4-6], and use keywords from the Wikipedia research fields’ corpus to assign a likelihood score that a given grant award’s title belongs to a given GSS research field category.

The results illustrated in Figure S2 are consistent with results shown in Figure 1: the marker for every field is well below the 45-degree line. In other words, although there are differences across fields, the likelihood of a woman on a team being named as an author on any given scientific document is uniformly and substantially lower across 91 of these more granular field definitions.

Figure S2: Women are less likely to be named in all fields, even with detailed categories


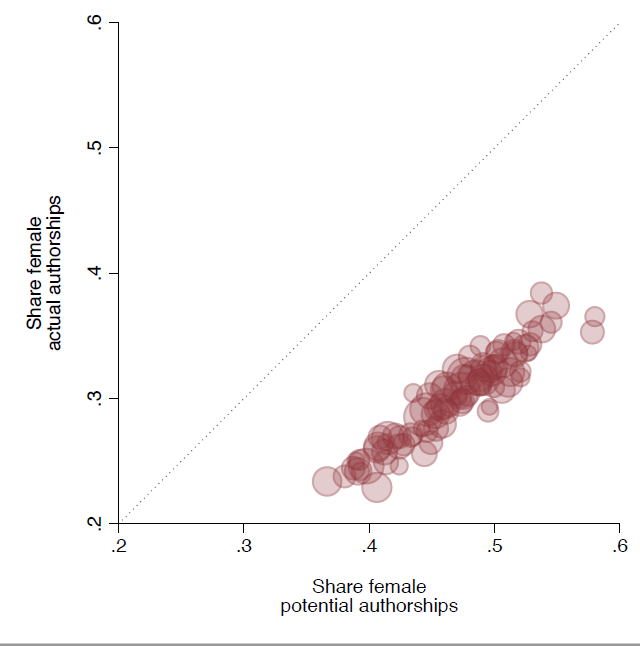


Note: The figure plots the probability a potential author on a scientific document (patent or article) is a woman (x-axis) against the probability an actual author is woman (y-axis) across fields. The markers in each panel are sized by the total number of actual authorships in the field category. The 45-degree line represents parity in the gender composition of potential and actual authorships.

## (vi). Sample Construction

Four questions regarding the sample construction were addressed using robustness checks.

The first question was whether the definition of potential authorship was too broad in its inclusion of all scientific documents associated with any of a team’s employees or grants. The check was to restrict potential authorships to those linked to articles and patents that include either the PI as an author or acknowledge the PI’s grants.

The second question was whether the one-year look-back window to be eligible for authorship was too restrictive. The check was to extend the period to two years, effectively expanding the risk set.^[[8]](#footnote-8)^

The third question was whether the focus should be on a subset of individuals who were linked to only one team. The baseline analyses used weights and adjusted standard errors for those individuals—particularly research staff and undergraduates—who appeared on multiple teams in the analytical sample. The check was to run the baseline specification on a subsample of individuals who are only associated with a single team.

The fourth question was the sensitivity to linkage quality. The check was to narrow the sample to include potential authors linked to a given article or patent only if they are explicitly linked to the grant acknowledged on that document.

Table S9 displays these results, where column 1 displays the baseline results, and columns 2-4 reflect the three robustness checks. In each of these robustness tests, the results are smaller in absolute terms but comparable in relative terms and statistically precise. Thus, the main result of the paper remains qualitatively unchanged in these more restrictive subsamples.

Table S9. Gender differences in attribution with different analytical samples

|  | (1) | (2) | (3) | (4) | (5) |
| --- | --- | --- | --- | --- | --- |
|  | Baseline | PI or Grant Acknowledged | 2-Year Eligibility | Affiliated with One Team | Employed on Acknowledged Grant |
|  |  | Articles |  |  |  |
| Woman | -0.00421^***^ | -0.00340^***^ | -0.00331^***^ | -0.00307^**^ | -0.01267^***^ |
|  | (0.00066) | (0.00066) | (0.00066) | (0.00151) | (0.00313) |
| N= | 17929271 | 12688230 | 13866427 | 719464 | 413642 |
| Dep. Mean | 0.032 | 0.030 | 0.030 | 0.041 | 0.124 |
|  |  | Patents |  |  |  |
| Woman | -0.00765^***^ | -0.00564^***^ | -0.00675^***^ | -0.00389^***^ | -0.03793^***^ |
|  | (0.00071) | (0.00069) | (0.00068) | (0.00102) | (0.00976) |
| N= | 3203831 | 2050180 | 2549747 | 228152 | 18843 |
| Dep. Mean | 0.013 | 0.012 | 0.012 | 0.009 | 0.090 |
| Calendar year x month | X | X | X | X | X |
| PI Flag | X | X | X | X | X |
| Days | X | X | X | X | X |
| Job Title | X | X | X | X | X |
| Field | X | X | X | X | X |
| Team | X | X | X | X | X |

Note: The observations are weighted by the inverse number of teams per employee times the inverse number of potential articles per employee. Controls for article calendar year x month, days worked in the team, a PI flag, job title, and an indicator for each team are included. Each coefficient is tested against the null hypothesis of being equal to 0 using a two-sided t-test. We do not adjust for multiple hypothesis testing. Standard errors are clustered by team and employee and are in parentheses. The statistical significance of each test is indicated by * p < 0.10, ** p < 0.05, *** p < 0.01.

## (vii). Research experience controls

All of the main specifications include a control for the number of days that the focal individual worked on a particular team. However, that variable is restricted to the 2013-2016 period and is truncated. The robustness of the estimates to a longer measure of research experience was checked by creating a variable that measures how long someone appears working in the overall UMETRICS data, beyond our sample period of 2013-16, for the maximum length of time available for each university in the data. The preferred set of models is estimated including this additional control variable as well as its square to provide additional flexibility given the greater variation of time employed. The results reported in Table S10 are very similar to the baseline estimates.

Table S10. Controlling for the total amount of time that people work beyond the sample period.

|  | | Baseline | (1) | (2) | (3) | (4) | (5) |
| --- | --- | --- | --- | --- | --- | --- | --- |
|  | Articles | | | | | | |
| Woman | | -0.00421^***^ | -0.01832^***^ | -0.01345^***^ | -0.00780^***^ | -0.00576^***^ | -0.00422^***^ |
|  | | (0.00066) | (0.00082) | (0.00070) | (0.00069) | (0.00069) | (0.00065) |
|  | |  |  |  |  |  |  |
| Total Days (beyond 2013-16) | |  | 0.00096^***^ | 0.00066^***^ | 0.00044^***^ | 0.00043^***^ | 0.00057^***^ |
|  | |  | (0.00003) | (0.00002) | (0.00002) | (0.00003) | (0.00002) |
|  | |  |  |  |  |  |  |
| Total Days ^2 | |  | -0.00000^***^ | -0.00000^***^ | -0.00000^***^ | -0.00000^***^ | -0.00000^***^ |
|  | |  | (0.00000) | (0.00000) | (0.00000) | (0.00000) | (0.00000) |
| N= | | 17929271 | 17929271 | 17929271 | 17929271 | 17929271 | 17929271 |
| Dep. Mean | | 0.032 | 0.032 | 0.032 | 0.032 | 0.032 | 0.032 |
|  | Patents | | | | | | |
| Woman | | -0.00765^***^ | -0.01466^***^ | -0.01252^***^ | -0.01001^***^ | -0.00889^***^ | -0.00762^***^ |
|  | | (0.00071) | (0.00077) | (0.00071) | (0.00070) | (0.00070) | (0.00070) |
|  | |  |  |  |  |  |  |
| Total Days | |  | 0.00033^***^ | 0.00023^***^ | 0.00010^***^ | 0.00012^***^ | 0.00014^***^ |
| (beyond 2013-16) | |  | (0.00003) | (0.00003) | (0.00003) | (0.00003) | (0.00003) |
|  | |  |  |  |  |  |  |
| Total Days ^2 | |  | -0.00000^*^ | -0.00000 | -0.00000 | -0.00000 | 0.00000 |
|  | |  | (0.00000) | (0.00000) | (0.00000) | (0.00000) | (0.00000) |
| N= | | 3203831 | 3203831 | 3203831 | 3203831 | 3203831 | 3203831 |
| Dep. Mean | | 0.013 | 0.013 | 0.013 | 0.013 | 0.013 | 0.013 |
| Calendar year x month | | X | - | X | X | X | X |
| PI Flag | | X | - | X | X | X | X |
| Job Title | | X | - | - | X | X | X |
| Field | | X | - | - | - | X | X |
| Team | | X | - | - | - | - | X |

Note: The observations are weighted by the inverse number of teams per employee times the inverse number of potential articles or patents per employee. Controls for article/patent calendar year x month, days worked in the team, a PI flag, job title, and an indicator for each team are included. Each coefficient is tested against the null hypothesis of being equal to 0 using a two-sided t-test. We do not adjust for multiple hypothesis testing. Standard errors are clustered by team and employee and are in parentheses. The statistical significance of each test is indicated by * p < 0.10, ** p < 0.05, *** p < 0.01.

## (vii). Logistic model

The estimates reported in the main text are based on linear probability models. This set of robustness checks re-estimates the model using logistic regressions. We omit the specifications that control for team (i.e. column 5 of Extended Data Table 4) because these models include a large number of fixed effects and are likely to create an incidental parameters problem as well as posed challenges in terms of convergence of maximum likelihood estimation. The coefficient estimates in Table S11 are presented as log odds ratios. The estimates are qualitatively similar, in terms of sign and statistical precision, to those reported in the main text.

Table S11. Gender Differences Estimated with a Logistic Regression

|  | (1) | (2) | (3) | (4) |
| --- | --- | --- | --- | --- |
| Articles | | | | |
| Woman | -0.66181^***^ | -0.51495^***^ | -0.30360^***^ | -0.22816^***^ |
|  | (0.01780) | (0.01778) | (0.01844) | (0.01882) |
| N= | 17929271 | 17929271 | 17929271 | 17929271 |
| Dep. Mean | 0.032 | 0.032 | 0.032 | 0.032 |
| Patents | | | | |
| Woman | -1.45005^***^ | -1.32035^***^ | -1.10600^***^ | -1.02999^***^ |
|  | (0.04992) | (0.04950) | (0.05053) | (0.05054) |
| N= | 3203831 | 3203831 | 3203831 | 3203831 |
| Dep. Mean | 0.013 | 0.013 | 0.013 | 0.013 |
| Calendar year x month | - | X | X | X |
| PI Flag | - | X | X | X |
| Days | - | X | X | X |
| Job Title | - | - | X | X |
| Field | - | - | - | X |
| Team | - | - | - | - |

Note: The observations are weighted by the inverse number of teams per employee times the inverse number of potential articles or patents per employee. Controls for article/patent calendar year x month, days worked in the team, a PI flag, job title, and an indicator for each team are included. Robust standard errors were calculated because it was not feasible to twoway cluster standard errors with a logistic regression. Statistical significance indicated by * p < 0.10, ** p < 0.05, *** p < 0.01.

***9. Combinations of robustness checks***

The above robustness checks are implemented one at a time, but combinations of robustness checks may yield different results. This section considers 7 robustness tests (examined individually in the previous sections) separately and in combinations of two and three. These include: (1) restricting the sample to people who were working on grants that were acknowledge on the article or patent (Table S9); (2) dropping people with Asian names (Table S1); (3) keeping only people with English names (Table S1); (4) restricting the sample to people who were linked to an ORCID, WoS Researcher ID for the article sample or a Patents View Inventor ID for the patent sample (Table S5); (5) restricting the sample to people who only appear in a single team (Table S9); (6) restricting the sample to articles / patents that include an employee’s PI as an author on the article (or inventor on the patent) or acknowledge one of her/his grants (Table S9); (7) dropping undergraduates and research staff (Table S5). Figure S3 shows the point estimates and confidence intervals from these robustness checks being run alone as well as in various two and three-way combinations. Two sample restrictions are excluded whenever they are in combination together: excluding Asian names and keeping only English names, because they overlap substantially. The baseline estimates from the main text are above the median (in magnitude) and hence conservative. Of the total 112 robustness tests examined across the two samples, only one two-way combination for the article sample (5 and 7 above) and one three-way combination for the patent sample (3, 4, and 5 above) had a coefficient estimate that was at or above zero. Even in these two extreme cases, the point estimates were not statistically significant, and the confidence interval extended into the negative range of possible values.

Figure S3. Combinations of Robustness Checks


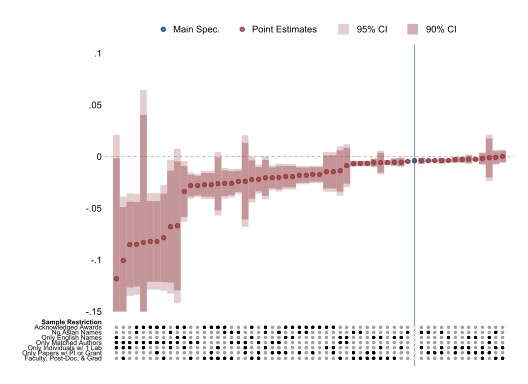

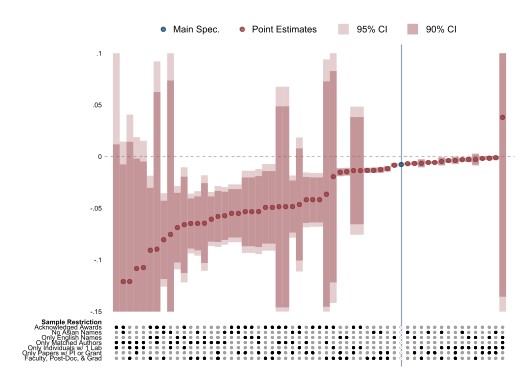


(a) (b)

Notes: For both the article sample (panel a) and patent sample (panel b), there were a total of 56 robustness tests conducted where 7 consisted of a single sample restriction, 20 consisted of combinations or two, and 29 consisted of combinations of three. We omit combinations of two and three sample restrictions which exclude Asian names and keep only English names since those restrictions are effectively the same.

# Part 2: Descriptive Statistics

## Information about the university sample

The final university sample consists 57 college campuses from 20 universities: Emory University, Indiana University, New York University, Northwestern University, Ohio State University, Oregon State University, Pennsylvania State University, Purdue University, Rutgers University, University of Arizona, University of California - San Diego, University of Illinois at Urbana-Champaign, University of Iowa, University of Kansas, University of Michigan, University of Missouri, University of Oregon, University of Texas – Austin, University of Wisconsin, Washington University in St. Louis.

In 2019, according to IPEDS, those campuses produced a total of 16339 doctorate recipients and employed 20,988 postdocs. 30 of the 36 universities had medical schools. 14 were land grant universities. The universities account for over 30% of university federal R&D expenditures; they were all classified with a Carnegie classification as: Doctoral Universities: Very High Research Activity.

## Characteristics of Matched Authors/Inventors

Most researchers in the sample are never named on an article or patent. Table S12 compares the full sample to those individuals who are ever matched to WoS or PatentsView in any year. Unsurprisingly, individuals who are matched work more days and are more likely to be faculty members. They are less likely to be women and more likely to have an Asian name.

Table S12. Characteristics of Matched Authors/Inventors

|  | (1) | (2) | (3) | (4) | (5) |
| --- | --- | --- | --- | --- | --- |
|  | Article (All) | Article  (Linked) | Article (Linked- ORCID or WoS ID) | Patent (All) | Patent (Linked) |
| Woman | 0.402 | 0.307 | 0.235 | 0.377 | 0.150 |
|  | (0.490) | (0.461) | (0.424) | (0.485) | (0.357) |
|  |  |  |  |  |  |
| Man | 0.430 | 0.518 | 0.575 | 0.449 | 0.659 |
|  | (0.495) | (0.500) | (0.494) | (0.497) | (0.474) |
|  |  |  |  |  |  |
| Days | 257.6 | 343.3 | 363.7 | 221.7 | 361.8 |
|  | (408.4) | (530.4) | (553.9) | (364.1) | (627.2) |
|  |  |  |  |  |  |
| Graduate | 0.242 | 0.294 | 0.242 | 0.242 | 0.183 |
|  | (0.422) | (0.448) | (0.422) | (0.423) | (0.381) |
|  |  |  |  |  |  |
| Post-Doc | 0.0862 | 0.127 | 0.168 | 0.0894 | 0.0842 |
|  | (0.276) | (0.325) | (0.364) | (0.280) | (0.270) |
|  |  |  |  |  |  |
| Faculty | 0.149 | 0.328 | 0.449 | 0.169 | 0.579 |
|  | (0.354) | (0.467) | (0.494) | (0.373) | (0.491) |
|  |  |  |  |  |  |
| Research Staff | 0.355 | 0.213 | 0.124 | 0.340 | 0.140 |
|  | (0.473) | (0.402) | (0.321) | (0.469) | (0.341) |
|  |  |  |  |  |  |
| Undergraduate | 0.169 | 0.0378 | 0.0165 | 0.159 | 0.0136 |
|  | (0.370) | (0.185) | (0.122) | (0.362) | (0.112) |
|  |  |  |  |  |  |
| English Name | 0.401 | 0.371 | 0.354 | 0.387 | 0.353 |
|  | (0.490) | (0.483) | (0.478) | (0.487) | (0.478) |
|  |  |  |  |  |  |
| Asian Name | 0.169 | 0.226 | 0.261 | 0.187 | 0.259 |
|  | (0.375) | (0.418) | (0.439) | (0.390) | (0.438) |
|  |  |  |  |  |  |
| Sample Size | 127241 | 37585 | 5623 | 71354 | 3439 |
|  |  |  |  |  |  |

## Funding Sources

The grants in the sample come from a variety of sources, both federal and nonfederal. Figure S4 illustrates the funding source distribution in the sample, at the grant level. The Department of Health and Human Services (HHS) is the largest funding source, for both men and women, but a variety of sources are represented in the data.

Figure S4: Distribution of Funding Sources


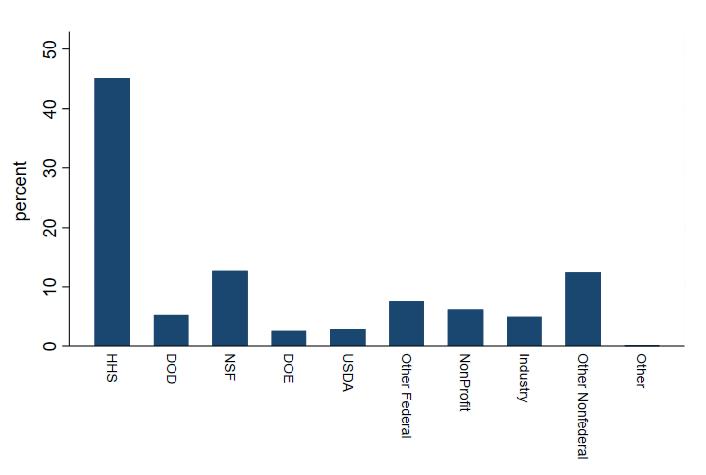


## Counts of Authors, Workforce, and Authorship

Tables S13 and S14 report the weighted and unweighted count of people in our sample by occupation, field, and authorship. The left panel of Table S13 reports the (weighted) number of ever authors overall and by gender pooling articles and patents across occupations and fields. The right panel reports the (weighted) number of actual authorships overall and by gender pooling articles and patents across occupations and fields. The ratio of these to the numbers in the left panel of Table S14 gives the rate of ever authors and the rate of authorship on a given document in ED Table 2 for men and women. The Total columns in ED Table 2 consider the full sample, including individuals to whom we were not able to assign a gender algorithmically.

The left panel of Table S14 reports the number of potential authors overall and by gender pooling articles and patents across occupations and fields. This corresponds to the headcount of people overall and in each occupation and field. The right panel reports the unweighted number of potential authorships overall and by gender pooling articles and patents.

Table S13: Counts of authors and authorships

|  | Number of Authors | | | Actual Authorship | | |
| --- | --- | --- | --- | --- | --- | --- |
|  | (1) | (2) | (3) | (4) | (5) | (6) |
|  | Total | Woman | Man | Total | Woman | Man |
| Overall | 18,034.00 | 6,284.00 | 11,750.00 | 3,443.77 | 1,096.57 | 2,347.20 |
| Grad Students | 4,320.50 | 1,348.69 | 2,971.82 | 582.41 | 196.91 | 385.50 |
| Postdocs | 2,069.33 | 693.42 | 1,375.92 | 322.80 | 105.26 | 217.53 |
| Faculty | 7,758.31 | 2,411.65 | 5,346.66 | 1,969.06 | 545.93 | 1,423.13 |
| Research Staff | 3,385.91 | 1,629.92 | 1,755.99 | 485.16 | 218.76 | 266.40 |
| Undergrad Student | 499.95 | 200.33 | 299.62 | 84.35 | 29.71 | 54.64 |
| Agriculture | 815.97 | 282.69 | 533.28 | 147.04 | 46.45 | 100.59 |
| Biology | 3,479.21 | 1,254.30 | 2,224.91 | 647.81 | 215.52 | 432.29 |
| Computer Sciences | 738.08 | 238.64 | 499.44 | 138.53 | 41.03 | 97.50 |
| Engineering | 1,848.97 | 554.41 | 1,294.56 | 322.88 | 90.71 | 232.17 |
| Geosciences | 945.47 | 283.25 | 662.23 | 181.22 | 49.08 | 132.14 |
| Health | 2,460.16 | 972.78 | 1,487.38 | 435.02 | 158.40 | 276.62 |
| Math | 676.53 | 219.07 | 457.46 | 141.52 | 39.68 | 101.83 |
| Multidisciplinary | 702.99 | 256.63 | 446.36 | 124.39 | 42.52 | 81.86 |
| Natural Resources | 767.46 | 271.24 | 496.22 | 141.68 | 45.61 | 96.08 |
| Physical Sciences | 1,378.39 | 393.93 | 984.45 | 261.63 | 67.90 | 193.73 |
| Psychology | 1,265.34 | 484.09 | 781.25 | 234.14 | 82.37 | 151.77 |
| Social Sciences | 1,681.39 | 618.58 | 1,062.81 | 304.90 | 102.74 | 202.17 |

Table S14: Counts of workforce and authorship

|  | Number of People (Headcount) | | | Potential Authorship | | |
| --- | --- | --- | --- | --- | --- | --- |
|  | (1) | (2) | (3) | (4) | (5) | (6) |
|  | Total | Woman | Man | Total | Woman | Man |
| Overall | 107,240.00 | 51,738.00 | 55,502.00 | 17,687,068.00 | 7,743,667.00 | 9,943,401.00 |
| Grad Students | 22,920.70 | 9,011.37 | 13,909.33 | 3,430,690.25 | 1,136,020.38 | 2,294,670.00 |
| Postdocs | 8,140.32 | 3,102.60 | 5,037.73 | 1,149,734.25 | 378,355.19 | 771,379.00 |
| Faculty | 16,790.10 | 5,846.30 | 10,943.81 | 4,944,506.50 | 1,532,548.25 | 3,411,958.25 |
| Research Staff | 40,680.27 | 24,736.31 | 15,943.96 | 6,680,389.50 | 3,997,501.00 | 2,682,888.25 |
| Undergrad Student | 18,708.61 | 9,041.43 | 9,667.18 | 1,481,747.63 | 699,242.13 | 782,505.50 |
| Agriculture | 4,859.23 | 2,296.54 | 2,562.69 | 857,450.56 | 366,166.06 | 491,284.50 |
| Biology | 17,736.92 | 8,564.58 | 9,172.34 | 3,221,319.25 | 1,459,041.50 | 1,762,277.75 |
| Computer Sciences | 4,381.88 | 1,989.80 | 2,392.08 | 753,772.13 | 312,248.88 | 441,523.25 |
| Engineering | 9,835.39 | 4,152.50 | 5,682.89 | 1,979,319.13 | 746,908.88 | 1,232,410.25 |
| Geosciences | 5,169.22 | 2,201.91 | 2,967.31 | 873,757.38 | 343,926.94 | 529,830.38 |
| Health | 14,068.67 | 7,472.40 | 6,596.27 | 2,438,420.25 | 1,170,737.38 | 1,267,682.88 |
| Math | 3,876.05 | 1,770.76 | 2,105.29 | 642,469.69 | 271,454.47 | 371,015.22 |
| Multidisciplinary | 4,543.36 | 2,288.95 | 2,254.41 | 743,181.75 | 334,043.69 | 409,138.09 |
| Natural Resources | 4,677.56 | 2,261.51 | 2,416.04 | 748,545.38 | 329,082.91 | 419,462.47 |
| Physical Sciences | 6,563.46 | 2,630.45 | 3,933.01 | 1,234,119.75 | 452,006.28 | 782,113.50 |
| Psychology | 7,775.26 | 4,069.93 | 3,705.33 | 1,250,727.88 | 588,995.13 | 661,732.75 |
| Social Sciences | 10,424.30 | 5,243.34 | 5,180.96 | 1,765,625.38 | 811,992.38 | 953,633.00 |

# Part 3: Survey and Interview Details

## Email Recruitment Script

Each respondent received the following email, where each individual’s name was embedded at the beginning of the email, and where each survey was personalized to that particular individual, all according to WoS records. Each email was sent from [bglennon@wharton.upenn.edu](mailto:bglennon@wharton.upenn.edu) through the Qualtrics survey.

*Dear [Insert Name],*

*We are inviting you to complete a 5-minute survey for a research project on your experiences regarding how scientific credit has been allocated in research teams in which you have worked.*

[*Please click here to complete the survey*](https://urldefense.com/v3/__https:/wharton.qualtrics.com/jfe/form/SV_3mD4D79L6Yddgge?Q_DL=OBr2s0j7M7v2q4X_3mD4D79L6Yddgge_MLRP_4VMj35Aco1BgHQy&Q_CHL=email__;!!IBzWLUs!BxyK8R7wqjKvzzRVblTtwHwFZiGAzIw0lN-NUEco3OC93nyFHOr2K-jekBYyFbQLKJjTLnM$)

*Or you can copy and paste the link below.*

[*https:wharton.qualtrics.com/jfe/form/SV_3mD4D79L6Yddgge?Q_DL=OBr2s0j7M7v2q4X_3mD4D79L6Yddgge_MLRP_4VMj35Aco1BgHQy&Q_CHL=email*](https://urldefense.com/v3/__https:/wharton.qualtrics.com/jfe/form/SV_3mD4D79L6Yddgge?Q_DL=OBr2s0j7M7v2q4X_3mD4D79L6Yddgge_MLRP_4VMj35Aco1BgHQy&Q_CHL=email__;!!IBzWLUs!BxyK8R7wqjKvzzRVblTtwHwFZiGAzIw0lN-NUEco3OC93nyFHOr2K-jekBYyFbQLKJjTLnM$)

*As you know, advancement in science relies on individuals being credited for scientific contributions. Even if you feel that you have never observed the allocation of scientific credit in research teams, we ask that you please complete this survey. It is important for us to hear your thoughts and opinions. If you have already received this email, we ask that you complete the survey only once.*

*This is research being conducted by Dr. Matthew Ross of Claremont College, Dr. Britta Glennon of University of Pennsylvania, Dr. Raviv Murciano-Goroff of Boston University, Dr. Enrico Berkes of The Ohio State University, Dr. Bruce Weinberg of The Ohio State University, and Dr. Julia Lane of New York University. This research is supported by a National Science Foundation grant 1932689 and approved by the University of Pennsylvania IRB.*

*Your participation is voluntary, and you may stop participating at any time. Feel free to answer as few or as many questions as you wish.*

*We are committed to protecting your identity. Any file that connects your name or email address to your survey responses will be password-protected and accessible only to me (Britta Glennon). De-identified and unedited responses may be shared with my coauthors.*

*Any personal information will be de-identified before article. After the study is complete, any files containing identifying information will be deleted.*

*The information collected by this survey is for academic research purposes only. No action will be taken based on information provided by you or any other individual responding to this survey.*

*If you have any questions about the survey, please contact me at* [*bglennon@wharton.upenn.edu*](mailto:bglennon@wharton.upenn.edu)*. We will send you the aggregate results when they are finalized.*

## Survey

The survey included three sections. In the first section, we identified the latest article that an individual’s ORCID had been listed on that was published between 2014 and 2018. In the context of that article, the survey confirmed authorship on that piece and asked each respondent what role they played in the conceptualization, management, analysis, and writing of that article. In the second section, respondents were asked if they had ever not been named on an article to which they had contributed in the past. For the respondents who indicated that they had been left off as an author of an article in the past, the survey asked the respondent to think about the most recent article in which they contributed to but had not been named as a coauthor. We asked what role they played in the work towards that article, as well as why they felt they had not been named as an author. The third and final section of the survey asked respondents about their demographic information, educational background, and work history. We included this section at the end of the survey in order to avoid priming effects and stereotype susceptibility.

Each respondent who clicked on the survey link was taken to the following welcome and instructions screen before proceeding to the survey itself. Each survey link is tailored to that individual, with their name and a article that they wrote embedded into the survey.

*Dear [embedded name],*

*Advancement in science relies on individuals being credited for scientific contributions. We are asking you to provide input into our research study on how scientific credit has been allocated in research teams in which you have worked.*

*If you agree to be in this study, we would like you to think about a paper that was produced by a research team in which you worked and answer a series of questions about that paper, followed by some more general questions. Information on the paper comes from public data sources, such as ORCID and the Web of Science. This work has been approved by the University of Pennsylvania IRB.*

*The survey will take fewer than 5 minutes.*

*We will send you the aggregate results when they are finalized. Your responses will, of course, be kept confidential.*

*If you have any questions about the survey, please contact Britta Glennon at bglennon@wharton.upenn.edu or the IRB at 215-898-2614.*

*---*

*Participation in this survey is completely voluntary. Feel free to answer as few or as many questions as you wish. We are committed to protecting your identity. Any file that connects your name or email address to your survey responses will be password-protected and accessible only to Britta Glennon. De-identified and unedited responses may be shared with coauthors. Any personal information will be de-identified before article. After the study is complete, any files containing identifying information will be deleted. De-identified responses will be kept for future research projects. The information collected by this survey is for academic research purposes only. No action will be taken based on information provided by you or any other individual responding to this survey.*

After clicking next, the respondent began the survey itself. Page 1 began by asking the respondent about a specific article identified from Web of Science.

*Q1a
Think about the paper "(Field/paper1title)," published in (Field/paper1journal) ((Field/paper1pubyear)).
How did you contribute to the paper? Check all that apply.
Note: Information about these categories can be found at*[*https:casrai.org/credit/*](https://casrai.org/credit/)

- *No role*
- *Conceptualization*
- *Data curation*
- *Formal Analysis*
- *Funding acquisition*
- *Investigation*
- *Methodology*
- *Project administration*
- *Resources*
- *Software*
- *Supervision*
- *Validation*
- *Visualization*
- *Writing – original draft*
- *Writing – review & editing*

*Q1b Were you listed as an author on the paper, "(Field/paper1title)"?*

- *Yes*
- *No*

*Q1c What was your occupation at the time that “(Field/paper1title)” was written?*

- *Graduate Student*
- *Post-Doc*
- *Faculty*
- *Research Staff*
- *Undergrad*
- *Other*
- *Prefer not to answer*

*Q1d Were you paid on a sponsored research grant while working on "(Field/paper1title)"?*

- *Yes*
- *No*
- *Don’t know*

Display the following questions if:

If Were you listed as an author on the paper, "(Field/paper1title)"? = No

And And Think about the paper "(Field/paper1title)," published in&nbsp;(e:Field/paper1journal) ((e:Field/paper1pubyear)). &nbsp; How did you contribute to the paper? Check all that apply.&nbsp;... q:QID8/SelectedChoicesCount Is Greater Than 0

And Think about the paper "(Field/paper1title)," published in (e:Field/paper1journal) (... != No role

*Q1e What is the most likely reason that you were not listed as an author?*

- *Contribution did not justify authorship*
- *Left the lab*
- *Personal responsibilities (e.g. maternity/paternity)*
- *Non-research professional responsibilities (e.g. department service, teaching, admin responsibilities, director of studies etc.)*
- *Some or all of the other members of the team underestimated my contribution*
- *Discrimination / Sterotyping / Bias*
- *Additional reasons (Open ended response)_______________________________*

*Q1f Please add additional comments if you wish*

*________________________________________________________________*

The next set of questions related to whether the respondent had ever contributed to a paper but not been named on it. Respondents who answered No to this question were taken directly to the demographics screen at the end. Respondents who answered Yes were asked a set of more detailed questions about their experience.

*Q2 Are there any papers to which you contributed but were not named as an author?*

- *Yes*
- *No*

Display the following Questions:

If Are there any papers to which you contributed but were not named as an author? = Yes

*Q2a Think of the most recent instance of a paper that you worked on and were not named as a coauthor on. How did you contribute to that paper? Check all that apply.*

- *No role*
- *Conceptualization*
- *Data curation*
- *Formal Analysis*
- *Funding acquisition*
- *Investigation*
- *Methodology*
- *Project administration*
- *Resources*
- *Software*
- *Supervision*
- *Validation*
- *Visualization*
- *Writing – original draft*
- *Writing – review & editing*

*Q2b What is the most likely reason that you were not listed as an author on that paper?*

- *Contribution did not justify authorship*
- *Left the lab*
- *Personal responsibilities (e.g. maternity/paternity)*
- *Non-research professional responsibilities (e.g. department service, teaching, admin responsibilities, director of studies etc.)*
- *Some or all of the other members of the team underestimated my contribution*
- *Discrimination / Stereotyping / Bias*
- *Additional reasons (Open ended response)_______________________________*

*Q2c What was your occupation at the time that the paper was written?*

- *Graduate Student*
- *Post-Doc*
- *Faculty*
- *Research Staff*
- *Undergrad*
- *Other*
- *Prefer not to answer*

*Q2d Was that paper written with support from a sponsored research grant?*

- *Yes*
- *No*
- *I don’t know*

*Q2e Roughly when was that paper written?*

*Q2f Please add additional comments about this or other similar experiences if you wish*

Finally, all respondents were asked a set of demographic questions and an open-ended response question.

*Q3 What field do you consider yourself in? (If your work is interdisciplinary please check the primary field.)*

- *Engineering*
- *Physical Sciences*
- *Environmental Sciences*
- *Mathematical Sciences*
- *Computer Science*
- *Life Sciences*
- *Psychology*
- *Social Sciences*
- *Other Sciences*
- *Arts / Humanities / Other*

*Q4 What is your age in years?*

*Q5 What is your gender?*

- *Male*
- *Female*
- *Non-binary / Fluid*
- *Other*
- *Prefer not to answer*

*Q6 Do you consider yourself:*

- *Hispanic or Latino or Spanish Origin of any race*
- *Not Hispanic or Latino or Spanish Origin*

*Q7 Do you consider yourself:*

- *American Indian or Alaska Native*
- *Asian*
- *Black or African American*
- *Native Hawaiian or Other Pacific Islander*
- *White*

*Q8 Where did you get your Bachelor’s degree?*

- *US*
- *Non-US*

*Q9 Have you been on other research teams in which individuals contributed to papers but were not named as authors? 

If so, please elaborate on the situation and why you think they weren’t named.*

*Q10 We are seeking individuals to interview over Zoom regarding their experiences with the allocation of credit in research teams.

If you would be interested in talking with us about your experiences, please enter your email below.

Your interview responses will be kept confidential.*

## Interview Protocol

The following protocol was used for the six follow-up interviews.

*Before doing anything: Change the interviewees name as soon as they log in. Make other interviewer co-host.*

**Brief introductions:**

It’s so nice to meet you, and we really appreciate your taking the time to speak with us. Just to quickly introduce myself, I’m Britta Glennon, I’m a professor at Wharton, and this is my coauthor [invite to self-introduce].

**IRB compliance script [read]:**

Before we get started, we want to touch base on a few things for IRB compliance.

As you know, this study is on the allocation of credit in research teams. If you agree to participate, we’ll be asking a series of questions about your personal experiences on research teams, which should last about 30 minutes. This is of course completely voluntary – you should feel free to answer as many questions as you wish.

You’ll see that we’ve changed your name. This is to protect your identity in any files that we save. We would like to record and transcribe this interview, but of course only with your consent. Your name will not be on the recordings or transcription, they will be password-protected, and only our team will see this information. We will not be releasing any personal information in the publication. If we want to use any anonymous quotes from the interview, we will ask you for permission first.

A quick overview of the risks and benefits. There is a risk of breach of confidentiality, but we will do everything possible to minimize that risk through anonymization. There are no direct benefits, but understanding how credit is distributed in research teams will be of significant benefit to society and the broader research community.

If you have any questions or concerns, please contact either [Insert relevant coauthor name] or myself, or either of our IRB offices, both of which have approved these interviews. Let me know if you need any of that contact information.

With that all being said, do you agree to participate?

**[WAIT for verbal consent]**

Do you agree to have this interview recorded/transcibed?

**[WAIT for verbal consent]**

**[Then start recording IF they consent]**

*On each of these push/follow up wherever possible. Key things that we want to know: (1) whether X experience is systematic (so need to prompt them to talk about an attribution experience). (2) If it’s related to personal characteristics (gender/race/etc…), but do NOT explicitly ask about any specific personal characteristic.*

QUESTION 1: On the last paper you wrote, how did you decide who was made an author?

- Was there anyone upset about those choices? How did you handle that?
- How does thinking about credit attribution differ for different levels of seniority?
- What do you think is best practice for determining attribution?

QUESTION 2: In your survey response, you shared a story about not receiving attribution [*Modify according to the individual]*. Could you share that story with us in more detail?

QUESTION 3: Can you think of any other example of at least one person (including you) who did not receive credit for a research contribution they made?

- If yes, why do you think they did not end up receiving credit? Was there any reason given?
- What was that person(s) / individual’s response to not receiving credit?

*Other questions/prompts:*

- Do you think this/these experience(s) is an outlier, or do you think it reflects a larger pattern?
- Please tell us anything else about your experience with attribution issues / the experience you witnessed.
- What can you tell us about the person / people who did not receive proper credit on the piece in terms of age, job position, seniority, gender, race, ethnicity, immigrant status, field?

# Part 4: Complementary View of Figures in Main Text

The following figures (S5-S8) provide a complementary view of Figures 1, 2, 4, and 5 in the main text. Specifically, they provide a visual summary of the precise datapoints underlying those main figures. Each figure is described in more detail below.

Figure S5 complements Figure 1 in the main text, which visualizes – in bubble chart form – the women’s share of potential authorships (x-axis) against the women’s share of actual authorship (y-axis). In Figure S5, the respective shares are reproduced as bar graphs, with the precise datapoints written above each bar, and where the women’s share of actual authorships is represented by the left, blue bar and the women’s share of potential authorships is represented by the right, red bar. As in Figure 1, subfigure (a) is distributed across occupation while subfigure (b) is distributed across fields.

Figure S5: Another view of Figure 1, Actual versus potential authorships by position (a) and field (b)


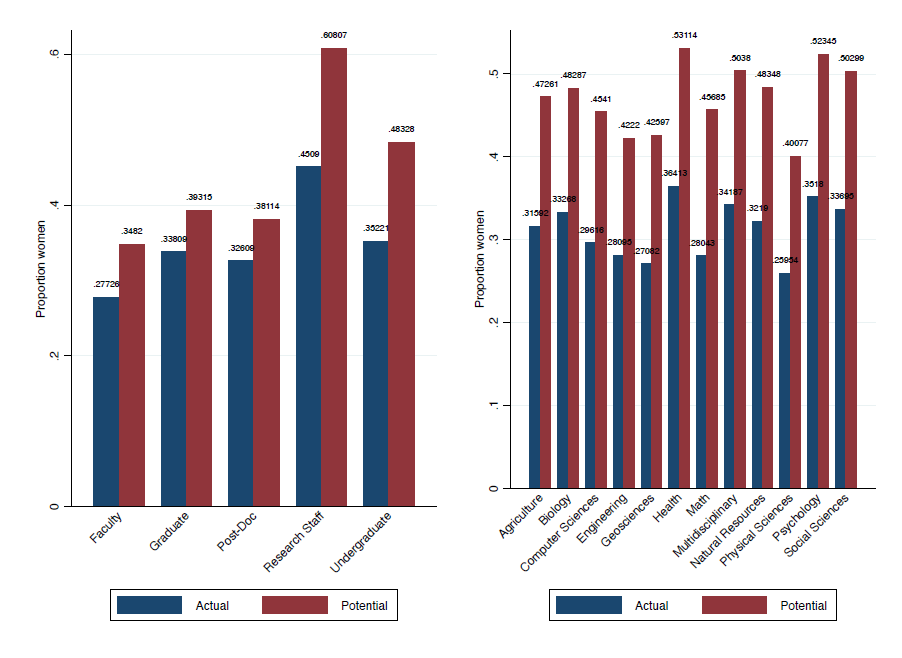


1. (b)

Note: This figure provides a visual summary of the data points for each category in Figure 1. The data are provided in Supporting Statistics tab Figure 1a and 1b.

Figure S6 complements Figure 2 in the main text. As in Figure 2, as one goes from left to right, more controls are steadily added; the final specification includes the full set of controls and fixed effects that are used as the baseline results in the remainder of the ppaer. As in Figure 2, the bars on the left represent the probability of being named for women while the bars on the right represent the probability of being named for men, and subfigure (a) represents articles while subfigure (b) represents patents. Here, we have added labels above each bar indicating the mean probability of being named for each gender.

Figure S6: Another view of Figure 2, Probability of women and men being named to articles (a) and patents (b)


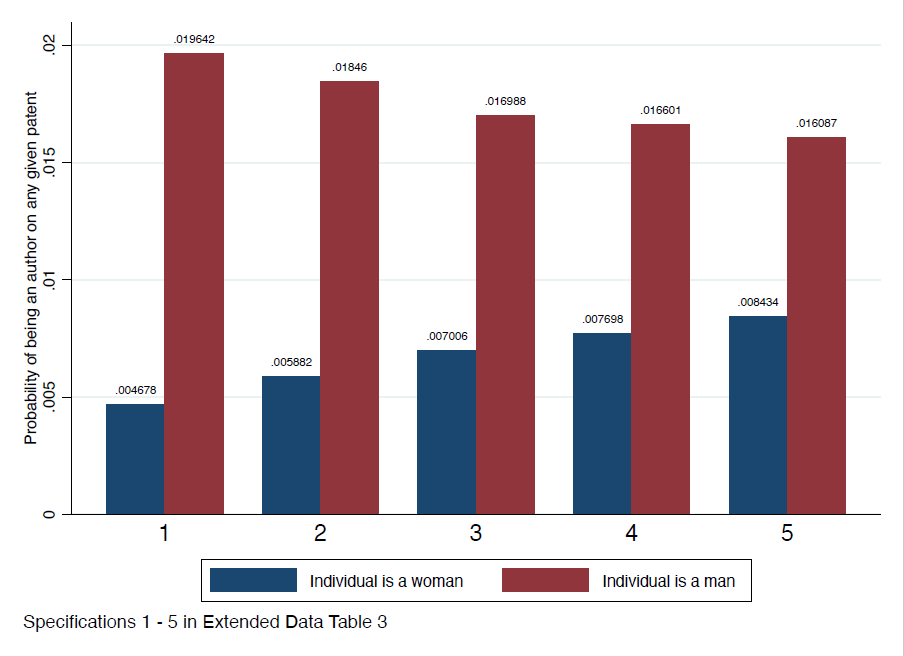

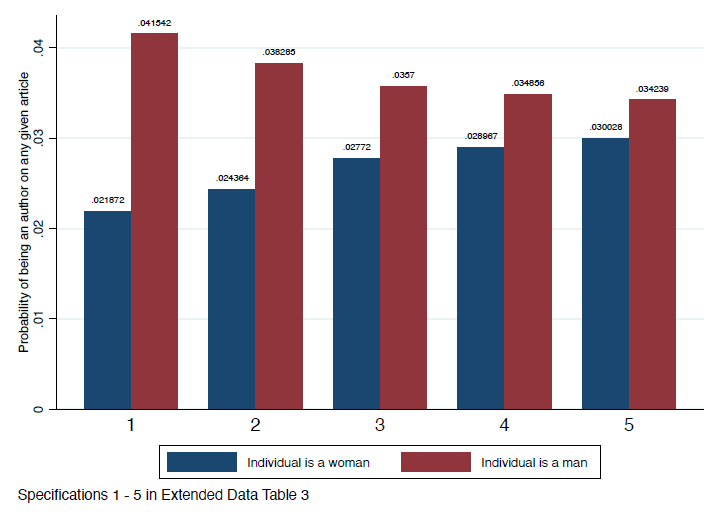


1. (b)

Note: This figure provides a visual summary of the mean data points for each category in Figure 2. The data are provided in Supporting Statistics tab Figure 2a and 2b

Figure S7 complements Figure 4 in the main text. As in Figure 4, it reports the response—by gender—to the survey question “What is the most likely reason that you were not listed as an author on that paper?”, where respondents could select more than one option. Specifically, it reports the share of women/men who gave any particular reason, with the precise datapoint listed above each bar.

Figure S7: Another view of Figure 4, Reason not named


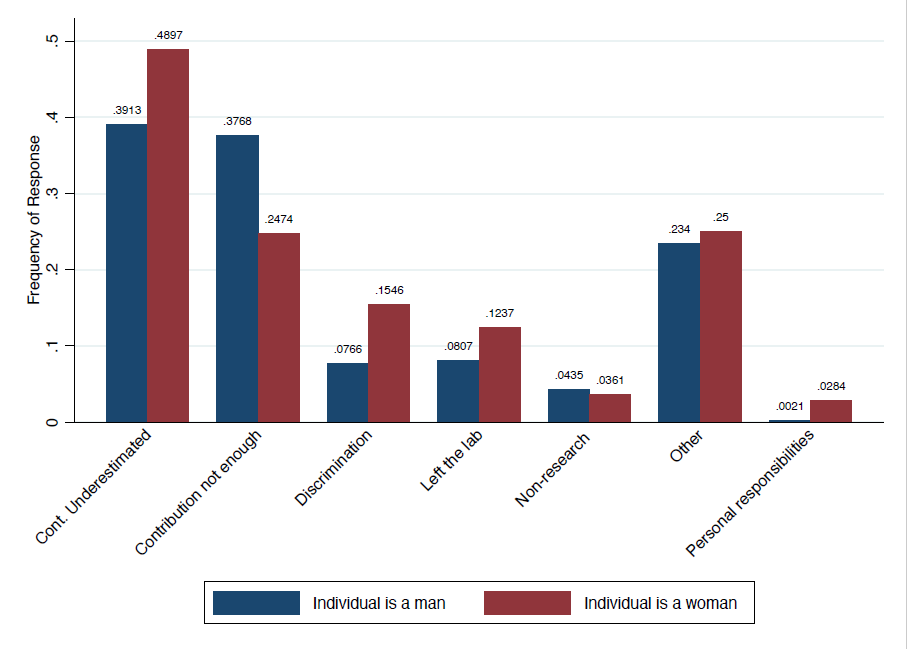


Note: This figure provides a visual summary of the mean data points for each category in Figure 4.

Figure S8 complements Figure 5 of the main text. As in Figure 5, it reports the response—by gender—to the survey question “How did you contribute to the [WOS-identified recent publication]? Check all that apply”, where respondents could select more than one option. The contribution options were identified by Project Credit. Figure S8 reports the share of women/men who indicated that they had contributed to a particular role, with the precise datapoint listed above each bar.

Figure S8: Another view of Figure 5, Contribution by Gender


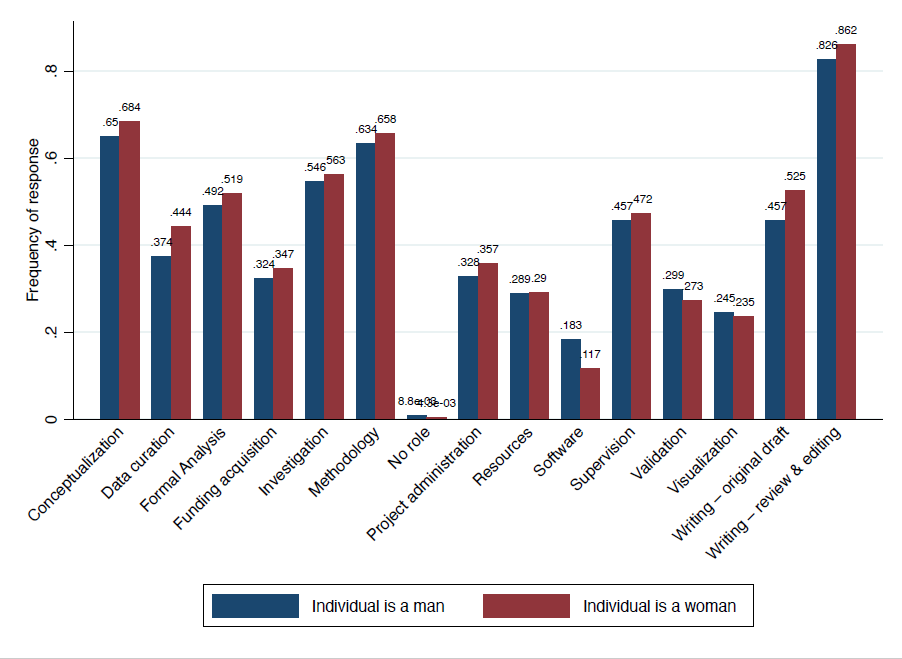


Note: This figure provides a visual summary of the mean data points for each category in Figure 5.

# References

1. Goldin, C. and M. Shim, *Making a name: Women's surnames at marriage and beyond.* Journal of Economic Perspectives, 2004. **18**(2): p. 143-160.

2. Fox, M.F., *Gender, family characteristics, and publication productivity among scientists.* Social Studies of Science, 2005. **35**(1): p. 131-150.

3. Mayer, S.J. and J.M. Rathmann, *How does research productivity relate to gender? Analyzing gender differences for multiple publication dimensions.* Scientometrics, 2018. **117**(3): p. 1663-1693.

4. Klochikhin, E. and P. Lambe, *A better way to classify science*, in *Research Fortnight*. 2015.

5. Levitskaya, E. and M. Ross, *Using Wikilabelling Techniques to Infer Research Fields from Text Data*, in *Coleridge Initiative Working Paper Series*. 2020, New York University: New York.

6. Nomoto, T. *Wikilabel: an encyclopedic approach to labeling documents en masse*. in *Proceedings of the 20th ACM international conference on Information and knowledge management*. 2011.

1. The random draw assigns genders based on a validation against the Survey of Earned Doctorates, where gender is known for those that the algorithm is unable to assign a gender. Approximately 60% are men and 40% are women. [↑](#footnote-ref-1)
2. It is worth noting, however, that the administrative data do not account for non-binary and non-dominant genders; such an analysis would be possible when universities and funding agencies begin to incorporate such information. [↑](#footnote-ref-2)
3. The frame for this analysis mimics the structure of, but was not drawn from, UMETRICS. The IRB protocol and the UMETRICS Data Use Agreement prohibits direct use of PII for such purposes. [↑](#footnote-ref-3)
4. ORCID is a unique persistent researcher identifier. [↑](#footnote-ref-4)
5. The appropriate comparison estimates are the ones obtained in column 3 of Table S5 in the subsequent section rather than column 5 of Extended Data Table 4. This is because both Table S5 and this analysis of rare/common names is restricted to those people in UMETRICS which have been linked to an ORCID or WoS Researcher ID. [↑](#footnote-ref-5)
6. In the full sample, matches also include names in WoS and PatentsView where the individual was not linked in each respective database to an author/inventor identity cluster, i.e., ORCID ID, WoS Researcher ID, or an Inventor ID. [↑](#footnote-ref-6)
7. A complete list of the *Web of Science* Document Types can be found here: [https:images.webofknowledge.com/WOKRS531OR13/help/WOK/hs_document_types.html](https://images.webofknowledge.com/WOKRS531OR13/help/WOK/hs_document_types.html) [↑](#footnote-ref-7)
8. We restricted articles and patents to 2015-2016 to ensure a full two-year look-back window and avoid truncation. [↑](#footnote-ref-8)
