# Supplementary material for: Women are credited less in science than men
Source: Nature. 2022 Jun 22;608(7921):135–45. doi: 10.1038/s41586-022-04966-w (PMC9352587; doi:10.1038/s41586-022-04966-w)
Supplement: Supplementary file 2 — Reporting Summary [file 41586_2022_4966_MOESM2_ESM.pdf]

Corresponding author(s): Julia Lane

Last updated by author(s): 5/28/2022

# Reporting Summary

Nature Portfolio wishes to improve the reproducibility of the work that we publish. This form provides structure for consistency and transparency in reporting. For further information on Nature Portfolio policies, see our Editorial Policies and the Editorial Policy Checklist.

## Statistics

For all statistical analyses, confirm that the following items are present in the figure legend, table legend, main text, or Methods section.

| n/a | Confirmed | |
|---|---|---|
| ☐ | ☒ | The exact sample size (*n*) for each experimental group/condition, given as a discrete number and unit of measurement |
| ☐ | ☒ | A statement on whether measurements were taken from distinct samples or whether the same sample was measured repeatedly |
| ☐ | ☒ | The statistical test(s) used AND whether they are one- or two-sided<br>*Only common tests should be described solely by name; describe more complex techniques in the Methods section.* |
| ☐ | ☒ | A description of all covariates tested |
| ☐ | ☒ | A description of any assumptions or corrections, such as tests of normality and adjustment for multiple comparisons |
| ☐ | ☒ | A full description of the statistical parameters including central tendency (e.g. means) or other basic estimates (e.g. regression coefficient) AND variation (e.g. standard deviation) or associated estimates of uncertainty (e.g. confidence intervals) |
| ☐ | ☒ | For null hypothesis testing, the test statistic (e.g. *F*, *t*, *r*) with confidence intervals, effect sizes, degrees of freedom and *P* value noted<br>*Give P values as exact values whenever suitable.* |
| ☒ | ☐ | For Bayesian analysis, information on the choice of priors and Markov chain Monte Carlo settings |
| ☐ | ☒ | For hierarchical and complex designs, identification of the appropriate level for tests and full reporting of outcomes |
| ☐ | ☒ | Estimates of effect sizes (e.g. Cohen's *d*, Pearson's *r*), indicating how they were calculated |

*Our web collection on statistics for biologists contains articles on many of the points above.*

## Software and code

Policy information about availability of computer code

**Data collection**

The UMETRICS data are hosted in the the Virtual Data Enclave at the University of Michigan. The data collection process from each university is described here https://iris.isr.umich.edu/wp-content/uploads/2022/01/new-member-handbook-2022.pdf Because the data are drawn directly from university HR and Finance systems, and each university can have different systems, the handbook notes "The task of compiling and transmitting administrative data from your HR, procurement, and research systems may feel
daunting. Some institutions have systems operating on very different platforms and are challenged at the thought of integrating disparate data sets, while others express concern about having to commit significant resources to compiling data.
At IRIS, we have worked with institutions that are quite diverse in how they manage data and we will walk through all of these issues with your data point of contact. Our technical director, Kevin Bjorne (kbjorne@umich.edu), has an outstanding record of helping institutions manage this process effectively. Kevin estimates the initial data transmission may take about 40 hours of institutional effort, and considerably less time for subsequent data transmissions. For institutions that participated in the federal STARMETRICS program this time can be much reduced by adapting existing scripts, as IRIS data are based on STARMETRICS data formats. Please contact us at IRIS-info@umich.edu to schedule an individual phone call or conference call to review the process if you have not done so already"

**Data analysis**

All the Stata code (Version 17) and Python 3.7.6 code used is available in the Virtual Data Enclave at the University of Michigan

For manuscripts utilizing custom algorithms or software that are central to the research but not yet described in published literature, software must be made available to editors and reviewers. We strongly encourage code deposition in a community repository (e.g. GitHub). See the Nature Portfolio guidelines for submitting code & software for further information.

# Data

Policy information about availability of data

All manuscripts must include a data availability statement. This statement should provide the following information, where applicable:

- Accession codes, unique identifiers, or web links for publicly available datasets
- A description of any restrictions on data availability
- For clinical datasets or third party data, please ensure that the statement adheres to our policy

The datasets generated during and/or analysed during the current study are available, as well as the associated code, at the Virtual Data Enclave repository at the Institute for Research on Innovation and Science at the University of Michigan. Access information is provided here https://iris.isr.umich.edu/research-data/access/ . Patent data were obtained from Patentsview (https://patentsview.org/), which is publicly available. Web of Science data were obtained from CADRE at Indiana University (https://iuni.iu.edu/resources/datasets/cadre).
The survey data are not available as per the University of Pennsylvania IRB protocols. Aggregate statistics from the survey data can be made available to researchers (upon request) for replication

# Human research participants

Policy information about studies involving human research participants and Sex and Gender in Research.

| | |
|---|---|
| Reporting on sex and gender | Sex and gender are the core of the analysis. Both males and females were studied, as well as those for whom no gender could be identified |
| Population characteristics | UMETRICS data: 128,859 individuals from 72 college and campuses who were paid on a grant in the period 2013-2016 from a participating institution. 51, 737 were female. 55,500 were male. Gender could not be determined for 21,622.<br><br>Survey: 2,446 Individuals who: (1) had a public profile on ORCID, (2) had an associated email address, and (3) published at least one academic paper in the Web of Science database between 2014 and 2018. 978 were female, 20 were fluid/ undefined gender, 1143 were male. The mean age was 49.72 years. 344 identified as Hispanic, 2,036 were White, 37 were Black, 356 were Asian, 17 were American Indian or Alaska Native / Native Hawaiian or Other Pacific Islander<br><br>Full details are presented in ED Tables 1, 2, 9, and 10. |
| Recruitment | For UMETRICS inclusion: All individuals who were paid on a research grant at participating institutions and whose data were provided by the institution were included in the study.<br>For survey inclusion:<br>We began by identifying individuals who had a public profile on ORCID, had an associated email address, and published at least one academic paper in the Web of Science database between 2014 and 2018. After adjusting for duplicates, there were 98,022 unique ORCID profiles that matched our sample criteria.<br><br>We ran three pilots that took samples of 500 individuals each that matched this criteria. We then stratified on gender for the main study, sampling 10,000 male, 10,000 female, and 6,500 gender-ambiguous names (based on the Ethnea database).<br><br>We emailed each of the individuals described above through the Qualtrics platform with a recruitment script and personalized email link (which incorporated information about the published article they were linked to through Web of Science). The full email text and survey information can be found in Section C of the Supplementary Online Materials.<br><br>Because our sample is based on those individuals who choose to respond to the survey, self-selection bias may exist. In particular, perhaps those who are most concerned about issues around attribution would be those most likely to choose to complete the survey. This could result in an inflated rate of respondents stating they have been left off of papers. However, since gender is not mentioned in the recruitment script, we do not expect this bias to differ across gender.<br><br>For interview inclusion:<br>338 individuals indicated on the survey that they would be open to an interview, and provided an email address. We selected six individuals among those 338 to interview.<br><br>For UMETRICS inclusion:<br>Inclusion in the UMETRICS database did not involve active recruitment. [INSERT MORE DETAIL] |
| Ethics oversight | University of Pennsylvania Institutional Review Board (IRB Protocol # 850522) approved the survey. University of Pennsylvania Institutional Review Board (IRB Protocol # 850522), Boston University Institutional Review Board (IRB Protocol #6412X) and the New York University Institutional Review Board (IRB Protocol #IRB-FY2022-6243) and the Ohio State University Institutional Review Board (IRB Protocol 2022E0133) approved the followup interviews. |

Note that full information on the approval of the study protocol must also be provided in the manuscript.

# Field-specific reporting

Please select the one below that is the best fit for your research. If you are not sure, read the appropriate sections before making your selection.

☐ Life sciences ☒ Behavioural & social sciences ☐ Ecological, evolutionary & environmental sciences

For a reference copy of the document with all sections, see nature.com/documents/nr-reporting-summary-flat.pdf

# Behavioural & social sciences study design

All studies must disclose on these points even when the disclosure is negative.

**Study description**

Both quantitative and qualitative. The quantitative component primarily relies on UMETRICS administrative data, constructing a potential attribution rate to a realized attribution rate within university administrative data, and how these rates differ by gender. The survey and interviews focused on the allocation of credit more broadly, with both quantitative components such as the roles on published papers, while the qualitative component had open-ended responses on the reasons behind not receiving credit.

**Research sample**

The UMETRICS dataset is constructed from three sources: internal Finance and Human Resources administrative data from 72 colleges and campuses, representing over 40% of total academic R&D spending in the United States, journal articles from the Web of Science and patent data derived from the universe of patents from the US Patent and Trademark Office (USPTO). The analysis focuses on a subset of 52 college campuses which consistently provided data for the period covering 2013-16. This restriction ensures that employment spells are long enough to reasonably identify PIs and teams as well as to observe the scientific documents produced by those teams from 2014-16. The full data include administrative level information from approximately 440,000 unique federal and non-federal awards, including approximately 23 million wage payments to about 650,000 deidentified individuals. The sample represents over 40% of federal funding made to academic institutions. The population of funding from non-federal (philanthropic, state, industry, and local) funding is unknown, so it is not possible to determine the representativeness of the non-federal portion of the funding. Similarly, the population of research teams is unknown, as is the population of individuals supported on research grants, so it is difficult to determine the representativeness of the UMETRICS sample. We are not aware of another large-scale dataset other than UMETRICS that could be used for this analysis.

The survey data was drawn from a sample of authors with ORCID IDs who recently published an academic article in Web of Science and had an associated email address. The sample was selected because of the personalized nature of the survey; each respondent received a personalized survey link to their email address, and the personalized survey included questions about a specific paper that they had published. The respondents to the survey overrepresented faculty members, women, and academics who received their Bachelor's degree outside of the US.

**Sampling strategy**

The UMETRICS data represent the universe of all transactions data for the campuses at participating institutions for the years in which they submitted data. Universities are recruited through consistent partnership with the Association of American Universities (www.aau.edu) and the Association of Public LandGrant Universities (www.aplu.org), as well as with the United Negro College Fund (https://Uncf.org) and Excelencia (https://edexcelencia.org). Details of membership are provided here https://iris.isr.umich.edu/membership/.

We subset the data to those campuses that consistently provided data for the period covering 2013-2016. Full details are available here https://iris.isr.umich.edu/research-data/2019datarelease/

For the survey, below is our calculation of the estimated sample size needed for two-sample comparison of proportions

Test Ho: p1 = p2, where p1 is the proportion in population 1
and p2 is the proportion in population 2
Assumptions:

alpha = 0.0500 (two-sided)
power = 0.8000
p1 = 0.2500
p2 = 0.3000
n2/n1 = 1.00

Estimated required sample sizes:

n1 = 1291
n2 = 1291

Based on the pilot samples (which drew a random sample), women composed a small proportion of the respondents to our survey. This is likely due to underrepresentation in the scientific academic community more generally. As a result, we stratified by gender for the main study: 10,000 women, 10,000 men, and 6,500 gender-ambiguous profiles were randomly selected to survey.

**Data collection**

The UMETRICS data are transactions data produced by each university. The information about how the data are produced, processed and standardized are here https://iris.isr.umich.edu/membership/for-current-members/

The survey data were collected through an online web-based (Qualtrics) survey. The full information is below
1. Target Population and Accrual:
The target population was researchers with scholarly publications. We accessed the target population through a sample of Web of Science published authors.

2. Key Inclusion Criteria:
All subjects must have published an article in a scholarly journal or have worked on an article that was eventually published.

3. Key Exclusion Criteria:
Not applicable

4 Subject Recruitment and Screening:
We emailed a sample constructed for our survey from public ORICD records and the Web of Science, as detailed below. The ORCID database contains CV-style information of millions of academic researchers. We use publicly available information that researchers have chosen to make public. Each ORCID record is associated with an ORCID ID, which is a unique identifier for the academic researcher.

We focused on the 897,264 ORCID records that listed a complete name in addition to at least one employment spell or at least one educational degree. We filtered these ORCID records to only those for which we have an associated email address (128,602). Because the ORCID database does not contain email addresses, we link to the Web of Science database, which contains e-mail addresses and the bibliometric information on a wide range of academic publications. Because the focus was on asking academic researchers about their experience with being named or not being named as coauthors on publications, the ORCID profiles were restricted to those that could be linked with a published academic paper in the Web of Science database between 2014 and 2018: 98,022 profiles fulfilled those criteria and were not duplicates.

5. Early Withdrawal of Subjects:
Participation was completely voluntary; all respondents could simply not complete the full survey and were informed that they can stop participating at any time.

6. Vulnerable Populations:
Not applicable

7. Populations vulnerable to undue influence or coercion:
Not applicable.

STUDY DESIGN:
We launched the survey after three pilots, which were doing using random samples of 500 names each. We sent out the survey with one follow-up reminder after one week. The survey was designed to gain a deeper understanding as to how credit is distributed, and whether that credit distribution varies for men and women. The survey was emailed out to respondents and was hosted on the Qualtrics platform. The survey was designed to take fewer than five minutes.

We followed up with one-on-one interviews if respondents indicated that they'd like to be contacted after the survey (in response to the final question on each survey: "We are seeking individuals to interview regarding their experiences with the allocation of credit in research teams. If you would be interested in talking with us about your experiences, please enter your email below. Your responses will be kept confidential."). The interviews occurred over Zoom for 30 minutes, and were recorded and transcribed in the instances when the respondent gave permission.

The data were analyzed at Britta Glennon's office at Wharton, and a de-identified and aggregated version of the data was shared with her co-authors at their institutions.

Julia Lane (at NYU) and Raviv Murciano-Goroff (at Boston University) also obtained IRB approval to conduct interviews with Britta Glennon.

| | |
|---|---|
| Timing | The UMETRICS data is 2013-2016; the publication and patent data (which are publicly available) go through 2019.<br>The Survey data collection began in January 2022 and concluded in April 2022. |
| Data exclusions | Not applicable. |
| Non-participation | Participation was completely voluntary; all respondents could simply not complete the full survey and were informed that they can stop participating at any time. |
| Randomization | NA |

# Reporting for specific materials, systems and methods

We require information from authors about some types of materials, experimental systems and methods used in many studies. Here, indicate whether each material, system or method listed is relevant to your study. If you are not sure if a list item applies to your research, read the appropriate section before selecting a response.

## Materials & experimental systems

| n/a | Involved in the study |
|---|---|
| ☒ | ☐ Antibodies |
| ☒ | ☐ Eukaryotic cell lines |
| ☒ | ☐ Palaeontology and archaeology |
| ☒ | ☐ Animals and other organisms |
| ☒ | ☐ Clinical data |
| ☒ | ☐ Dual use research of concern |

## Methods

| n/a | Involved in the study |
|---|---|
| ☒ | ☐ ChIP-seq |
| ☒ | ☐ Flow cytometry |
| ☒ | ☐ MRI-based neuroimaging |
